# Recent Advances in Peptide-Loaded PLGA Nanocarriers for Drug Delivery and Regenerative Medicine

**DOI:** 10.3390/ph18010127

**Published:** 2025-01-18

**Authors:** Hossein Omidian, Renae L. Wilson, Ana M. Castejon

**Affiliations:** Barry and Judy Silverman College of Pharmacy, Nova Southeastern University, Fort Lauderdale, FL 33328, USA; rw1273@mynsu.nova.edu (R.L.W.); castejon@nova.edu (A.M.C.)

**Keywords:** peptide therapeutics, PLGA nanocarriers, controlled drug release, regenerative medicine, targeted drug delivery

## Abstract

Peptide-loaded poly(lactide-co-glycolide) (PLGA) nanocarriers represent a transformative approach to addressing the challenges of peptide-based therapies. These systems offer solutions to peptide instability, enzymatic degradation, and limited bioavailability by providing controlled release, targeted delivery, and improved stability. The versatility of PLGA nanocarriers extends across therapeutic domains, including cancer therapy, neurodegenerative diseases, vaccine development, and regenerative medicine. Innovations in polymer chemistry, surface functionalization, and advanced manufacturing techniques, such as microfluidics and electrospraying, have further enhanced the efficacy and scalability of these systems. This review highlights the key physicochemical properties, preparation strategies, and proven benefits of peptide-loaded PLGA systems, emphasizing their role in sustained drug release, immune activation, and tissue regeneration. Despite remarkable progress, challenges such as production scalability, cost, and regulatory hurdles remain.

## 1. Peptide-Loaded PLGA Therapeutics

Peptide-based therapeutics are advancing rapidly due to their high specificity, potency, and wide applicability across various diseases, including cancer, infectious diseases, and neurodegenerative disorders. Peptides are short chains of amino acids connected by peptide bonds, typically comprising two to fifty amino acids. They serve as versatile bioactive molecules in various therapeutic applications, including antimicrobial therapy, immunomodulation, tissue regeneration, and hormonal analogs. Their high specificity and biocompatibility make them attractive therapeutic candidates, but their clinical utility is limited by inherent challenges such as poor bioavailability, susceptibility to enzymatic degradation, and difficulties in targeted delivery [1,2,3,4,5,6,7,8]. These limitations compromise therapeutic efficacy and emphasize the critical need for innovative delivery systems to protect peptides and ensure their functional stability.

Poly(lactide-co-glycolide) (PLGA) has emerged as a leading material for drug delivery due to its biodegradability, biocompatibility, and flexibility in modulating drug release profiles [9,10,11]. These properties make PLGA ideal for peptide encapsulation and delivery. By offering solutions to peptide instability, enzymatic degradation, and limited bioavailability, PLGA-based systems enable controlled release, protection against degradation, and enhanced biodistribution. For instance, sustained and localized drug delivery provided by PLGA nanocarriers mitigates systemic side effects while preserving peptide bioactivity [12,13,14]. Such systems have demonstrated their ability to overcome peptide-specific challenges, including ensuring consistent therapeutic concentrations and mitigating rapid clearance or degradation in vivo [9,15,16,17,18,19]. This strategic integration of peptide therapeutics into advanced delivery platforms enhances their clinical application, offering a robust response to the limitations of traditional peptide therapies.

Peptides can be classified based on their origin into natural, synthetic, or recombinant categories. Natural peptides are derived from the enzymatic hydrolysis of proteins and include examples like the antihypertensive peptide KGYGGVSLPEW obtained from whey protein [16]. Microbial sources contribute antimicrobial peptides such as Dermaseptin-PP, which is used for wound-healing applications [20]. Synthetic peptides, on the other hand, are designed and engineered for specific purposes, such as G17 and G19 peptides for combating resistant bacterial infections like Methicillin-resistant Staphylococcus aureus (MRSA) and *E. coli* [21]. Recombinant DNA technology enables the large-scale production of therapeutic peptides, exemplified by recombinant Interleukin-10 (IL-10) and myelin oligodendrocyte glycoprotein (MOG) peptides for treating autoimmune encephalomyelitis [15].

Structurally, peptides may be linear, such as antimicrobial peptides like OH-CATH30 used for bacterial keratitis treatment [17], or cyclic, such as somatostatin analogs for long-term cancer therapy [22]. Functionally, peptides serve various roles. Antimicrobial peptides, such as LL37, are known for their ability to enhance wound healing [23], while immunomodulatory peptides, such as ESAT-6(1-20), are employed in vaccine development for tuberculosis [24]. Additionally, hormonal peptides like leuprolide acetate are used for treating prostate cancer and other hormone-related conditions [1]. Peptides also play a pivotal role in regenerative medicine, as seen with BMP-2-derived P24 peptides for bone tissue repair [18].

The versatility of peptide-loaded PLGA systems spans diverse therapeutic domains. For neurological disorders, PLGA nanocarriers facilitate the delivery of therapeutic agents across the blood/brain barrier (BBB), a significant challenge in treating conditions such as Alzheimer’s disease and glioblastoma [25,26,27,28,29]. The incorporation of peptides such as cyclic beta-hairpin BSBP8 for inhibiting amyloid beta aggregation further underscores their potential in addressing neurodegenerative diseases [27].

In vaccine delivery, sustained antigen release and immune system activation achieved through PLGA systems play a pivotal role in eliciting robust and durable responses [24,30,31,32,33]. The use of antigenic peptides, such as ESAT-6(1-20) for tuberculosis vaccines, highlights the ability of PLGA carriers to enhance vaccine efficacy [24]. In cancer therapy, peptide-functionalized PLGA nanoparticles, such as those targeting hepatocellular carcinoma with SP94 peptides, offer precise delivery to tumor sites, minimizing off-target effects and improving therapeutic outcomes [34,35].

Regenerative medicine represents another frontier for peptide-loaded PLGA systems. By leveraging their ability for controlled peptide release, these systems support critical processes such as bone regeneration, wound healing, and nerve repair. Hybrid formulations and functionalized scaffolds further enhance functionality by combining therapeutic and structural roles, extending their utility in tissue engineering applications [18,19,36,37,38]. For example, PLGA systems functionalized with BMP-2-derived peptides have been shown to support osteointegration and enhance the healing of bone defects [19,39].

Despite these advancements, peptide-loaded PLGA systems face challenges in scalability, reproducibility, and cost-effectiveness in large-scale production [40,41,42]. Addressing these hurdles requires continued innovation in polymer chemistry, nanotechnology, and pharmaceutical engineering to optimize these platforms for broader applications and clinical accessibility [14,43,44]. By combining peptide engineering with advancements in nanotechnology, future research can further improve their stability, efficacy, and patient-specific customization. These developments are expected to drive significant progress in personalized medicine and open new avenues for peptide-based therapies [9,15,20].

## 2. Therapeutic Roles of Peptides and Drugs in PLGA Systems

PLGA-based systems are redefining therapeutic strategies across various medical domains by enabling the targeted delivery and controlled release of bioactive agents. Their applications span cancer treatment, neurodegenerative disease management, tissue regeneration, infectious disease control, and advanced gene delivery systems.

### 2.1. Cancer Therapy

PLGA nanocarriers have transformed cancer therapy by improving the delivery and effectiveness of therapeutic agents. For instance, leuprolide acetate, encapsulated in PLGA, provides sustained treatment for prostate cancer by modulating hormonal pathways [1]. Functionalized nanoparticles, such as T7 peptide-functionalized carmustine systems, enhance blood/brain barrier (BBB) penetration, addressing challenges in glioma therapy [25].

**Figure 1:**
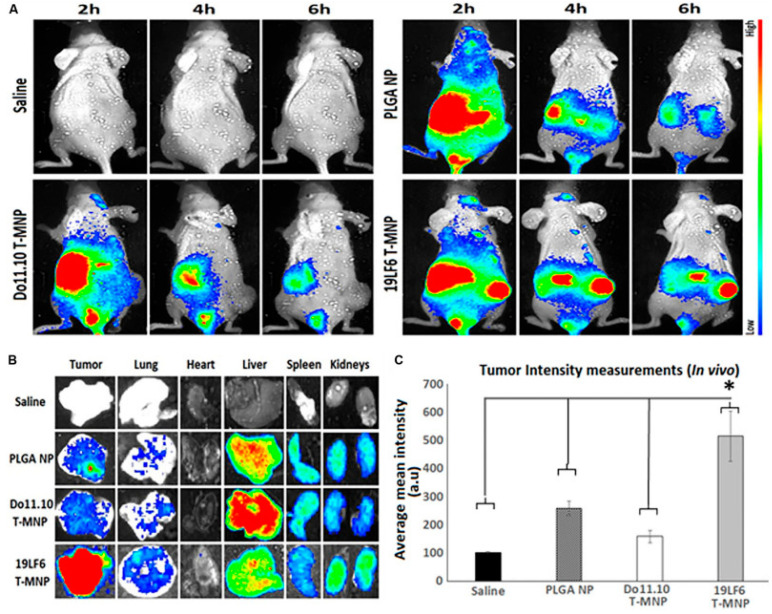
In vivo and ex vivo analysis of T-MNP biodistribution. (**A**) Characteristics of the IV-injected NPs on melanoma tumor models with real-time tumor targeting at 2 h, 4 h, and 6 h time intervals. (**B**) Images of the ex vivo organ biodistribution in different study groups. (**C**) Intensity of in vivo biodistribution study groups in tissue homogenates measured via fluorescent (*n* = 6 per group). * Statistically significant with *p* < 0.05. Poly-lactide-co-glycolide (PLGA); Nanoparticle (NP); T-cell membrane-coated PLGA NPs (T-MNPs); DO10.11 membrane-coated PLGA NPs (D-MNP’s); Coumarin-6 (C-6); A549 membrane-coated PLGA NPs (A-MNP’s); Naked nanoparticle (NNP); Optical Density (OD); Inhibitory Concentration (IC); Reverse Transcriptase Polymerase Chain Reaction (RT-PCR); Ultraviolet–Visible (UV–Vis); 3-(4,5-dimethylthiazol-2-yl)-5-(3-carboxymethoxyphenyl)-2-(4-sulfophenyl)-2H-tetrazolium (MTS). Adopted with permission [47].

Immunotherapeutic strategies have also advanced with synthetic long peptides (SLPs) and antigenic peptides, which activate cytotoxic T-cell responses, offering promising pathways for cancer immunotherapy [33,45]. Dual-drug delivery systems, including doxorubicin paired with anti-PD-L1 peptides, combine chemotherapy and immunotherapy for enhanced tumor suppression [46]. Similarly, Trametinib-loaded nanoparticles, cloaked with melanoma-specific T-cell membranes, demonstrate superior tumor-targeting capabilities (Figure 1) [47].

### 2.2. Neurodegenerative Disease Management

Peptide-loaded PLGA systems have demonstrated significant potential in neurodegenerative disease therapy. For Alzheimer’s disease, curcumin functionalized with Tet-1 or B6 peptides reduces amyloid plaques, oxidative stress, and neuroinflammation [3,5]. Insulin encapsulated in CPP-modified PLGA nanoparticles shows promise for treating cognitive dysfunction via intranasal delivery, bypassing the BBB [29]. Innovations like berberine conjugated with Tet-1 to reduce Tau phosphorylation [8] and phytol-loaded nanoparticles for disrupting amyloid aggregates [48] further enhance therapeutic options for complex neurological disorders.

### 2.3. Tissue Regeneration and Bone Repair

**Figure 2:**
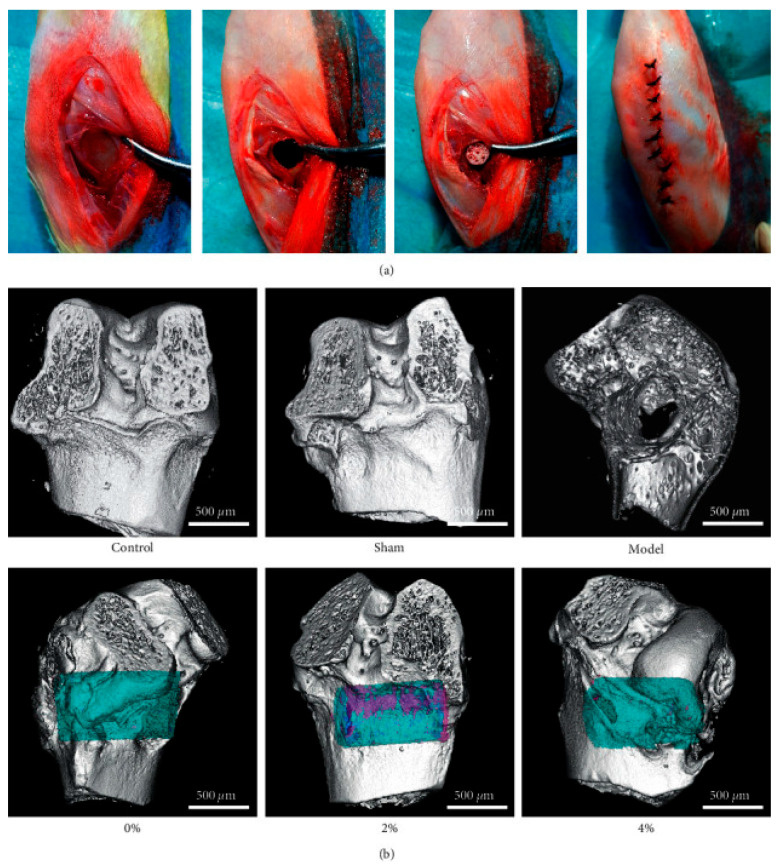
A 3D-printed PLGA scaffold composite peptide hydrogel implant with BMP-9 and P-15 in the treatment of bone defects in rabbits. (**a**) Implantation in rabbit bone process. (**b**) MicroCT test used for the detection of new bone formation and tissue-related protein expressions (blue, scaffold; pink, new bone). The MicroCT detection of 2% polypeptide scaffold showed good bone repair, promoting the expression of ALP, COL-1, OCN, RUNX2, and Sp7. Adopted with permission [38].

PLGA-based systems incorporating bioactive peptides significantly advance bone regeneration and tissue repair. For example, BMP-2 delivered via PLGA-RADA16 hydrogels or collagen-mimetic peptides enhances osteogenesis in bone defect models [19,38]. Additionally, adrenomedullin and PTHrP1-34 peptides promote osteoblast proliferation and angiogenesis, accelerating tissue repair [49,50]. In neural tissue repair, RADA16-I-BMHP1 peptides embedded in nanofibers support Schwann cell differentiation, fostering peripheral nerve regeneration [51,52]. Figure 2 shows in vivo experiments with the use of 3D-printed PLGA scaffolds with BMP-9 and P-15 peptide hydrogel in the treatment of bone defects in rabbits [38].

### 2.4. Infectious Diseases and Vaccines

PLGA systems have shown immense potential in combating infectious diseases and advancing vaccine technology. Antimicrobial peptides like SAAP-148 and OH-CATH30 effectively target multidrug-resistant pathogens and bacterial keratitis [9,17]. Vaccine candidates, including W-1 L19 peptides for canine parvovirus and multi-epitope peptides for influenza, elicit robust immune responses [53]. In response to COVID-19, oseltamivir phosphate encapsulated with SBP1 peptides targets the SARS-CoV-2 spike protein, offering potential therapeutic benefits [54].

### 2.5. Wound Healing

Peptide-functionalized PLGA nanoparticles accelerate wound healing by promoting angiogenesis, granulation, and collagen deposition. LL37 peptides enhance wound closure while providing antimicrobial benefits [23]. BMP-2-loaded PLGA nanoparticles combined with RADA16 hydrogels support osteogenesis and tissue regeneration in wound models [19]. Additionally, MSI-78(4-20), a potent antimicrobial peptide, combats infections and expedites recovery [55].

### 2.6. Gene Delivery and Genetic Therapy

PLGA nanoparticles functionalized with cell-penetrating peptides or RGD ligands enhance the intracellular delivery of genetic materials. DNA-loaded nanoparticles targeting lung epithelial cells enable effective therapeutic gene expression, with implications for treating genetic disorders [56,57]. Furthermore, short cationic peptide nucleic acids (PNAs) encapsulated in PLGA nanoparticles provide a promising platform for antisense and gene-editing applications [58].

### 2.7. Combating Infections

Incorporating antimicrobial peptides into PLGA systems offers effective strategies against bacterial infections. Peptides like G17 and G19 target resistant strains, including MRSA and E. coli, while ponericin G1 and bFGF contribute to wound healing and antibacterial action [21,59]. Fusion peptides that combine antimicrobial, osteogenic, and angiogenic properties address bone defect repair and infection control [60].

### 2.8. Additional Applications

Peptide-loaded PLGA systems have shown versatility in addressing unique medical needs. For example, Asiatic acid functionalized with renal-targeting peptides enhances kidney-specific drug delivery for chronic kidney disease [61]. In glioma treatment, dual-peptide systems such as Euphorbia factor L1 improve tumor targeting by crossing the BBB [62]. SDF-1alpha and VEGF peptides co-loaded in PLGA scaffolds promote angiogenesis and skin regeneration, supporting diabetic wound healing [37].

Table 1 explores the diverse therapeutic applications of peptide-loaded PLGA systems, categorizing active ingredients based on their clinical use, such as antimicrobial, hormonal, and anticancer therapies. These systems address critical challenges, including instability and poor bioavailability, while enabling targeted delivery and immune activation. Patterns reveal their extensive utility in combating antimicrobial resistance, facilitating vaccine development, and advancing regenerative medicine. The data highlight PLGA’s capacity to stabilize peptides, improve targeting precision, and reduce dosing frequency, showcasing its pivotal role in modern therapeutic strategies.

## 3. Essential Polymers and Additives in Peptide-Loaded PLGA Systems

**Figure 3:**
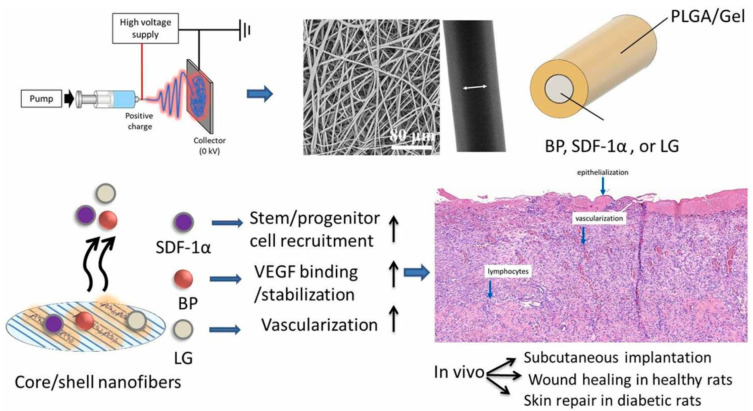
Shows stromal cell-derived factor-1α (SDF-1), vascular endothelial growth factor (VEGF)-binding peptide (BP), glucagon-like peptide-1 analog (GLP), and liraglutide (LG) in core/shell poly(L-lactide-co-glycolide)/gelatin fibers used to harness synergistic effects for skin repair in healthy and diabetic wound models in rats. Adopted with permission [37].

PLGA polymers serve as the foundation for numerous peptide-loaded delivery systems due to their excellent biodegradability and ability to provide controlled drug release. Adjustments to PLGA, such as incorporating acid- or ester-terminated variants, enable precise manipulation of its degradation properties and peptide release kinetics in specific environments [10,43]. To enhance solubility and biocompatibility, PLGA is frequently combined with polyethylene glycol (PEG) or its grafted derivatives, resulting in PEG/PLGA polymers that improve both peptide stability and targeting capabilities [35,74,82]. Moreover, hybrid composites blending PLGA with polymers like polycaprolactone (PCL) or gelatin-based hydrogels further expand their use, particularly in neural and skeletal tissue engineering applications [37,85,86]. Figure 3 illustrates the use of co-axial PLGA–gelatin fibers containing stromal cell-derived factor-1α (SDF-1α) and angiogenic signals for enhancing cutaneous wound healing [37]. Additionally, porous PLGA has been adopted in vaccine formulations and tissue-targeted delivery systems to enhance payload encapsulation and enable site-specific release [87].

The integration of peptides into PLGA-based systems offers distinct therapeutic advantages and facilitates precise tissue targeting. For example, cell-penetrating peptides (CPPs) such as TAT, R8, and SBP1 enable effective drug delivery across challenging biological barriers like the blood/brain barrier or intestinal epithelium, significantly improving bioavailability for diseases such as Alzheimer’s and diabetes [28,29,39]. Bioactive peptides, including RGD and YIGSR, play a vital role in promoting skeletal muscle differentiation, while BMP-2 and P-15 peptides are crucial for bone regeneration and repair [19,38,88]. Other specialized peptides, such as OH-CATH30 and MSI-78(4-20), exhibit antimicrobial properties, whereas angiogenic peptides like VEGF and Apelin contribute to wound healing and tissue regeneration [55,59,89].

Surface modifications of PLGA nanoparticles are essential for enhancing stability, targeting, and bioactivity. Coatings with PEG or polyethyleneimine (PEI) effectively prevent aggregation, extend circulation times, and enable applications such as gene delivery [3,90]. Polydopamine coatings promote osteogenic differentiation and improve mechanical integration for bone-related applications, while graphene oxide enhances hydrophilicity and mechanical strength for skeletal tissue engineering [59,91,92]. Functionalization with erythrocyte membranes or maleimide/PEG facilitates immune evasion and ensures stable conjugation, critical attributes for cancer therapeutics [62,82]. These modifications not only extend the circulation lifespan of nanoparticles but also optimize their interaction with target tissues.

Additives are integral to preserving peptide bioactivity and stabilizing PLGA formulations. Materials such as carboxymethyl chitosan (CMCS) and calcium phosphate (Ca_3_(PO_4_)_2_) are commonly used to prevent peptide acylation, a process that can degrade peptide integrity during PLGA breakdown [93,94]. Stabilizers, such as hydroxypropyl-beta-cyclodextrin, protect sensitive peptides like peptide-24, ensuring sustained therapeutic efficacy in bone regeneration applications [95]. Superparamagnetic iron oxide nanoparticles (SPIONs) are incorporated into PLGA systems to enable simultaneous imaging and therapeutic delivery, exemplifying the multifunctionality of these nanoparticles [96]. Fluorapatite further enhances the bioactivity of PLGA formulations, particularly in dental and bone-related applications [97].

Innovative materials also contribute to the versatility of PLGA-based systems. Dopamine ad-layers enhance scaffold adhesion, promoting osteointegration and cellular proliferation [91,92]. Self-immolative-protecting groups safeguard peptides during formulation processes, thereby maintaining their functional stability under challenging conditions [98]. Additionally, fluorescent dyes like Rhodamine-B are used to enable the tracking and imaging of nanoparticles in cellular delivery studies [28,99].

Table 2 highlights the pivotal role of PLGA as a versatile nanocarrier, enhanced by additives like PEG, CPPs, and adjuvants to address specific therapeutic challenges. PEG improves circulation and biocompatibility, while CPPs enable intracellular and barrier-crossing delivery, crucial for CNS and cancer treatments. Adjuvants like CpG amplify vaccine responses, and hydroxyapatite supports bone-targeted delivery. Innovations in self-assembling peptides (e.g., RADA16) bolster regenerative applications. Sustained-release strategies, leveraging stabilizers and ion-pairing agents, extend peptide bioavailability. The integration of these excipients maximizes efficacy in applications like antimicrobials, gene therapy, and vaccines, offering a robust blueprint for tailored peptide-loaded PLGA systems targeting a wide range of diseases.

## 4. Methodologies and Optimization Strategies for Peptide-Loaded PLGA Products

Peptide-loaded PLGA systems are critical for advanced drug delivery and tissue engineering applications. This section consolidates methodologies and optimization strategies and details techniques and guidelines for producing high-performance systems.

### 4.1. Emulsion Solvent Evaporation Techniques

**Figure 4:**
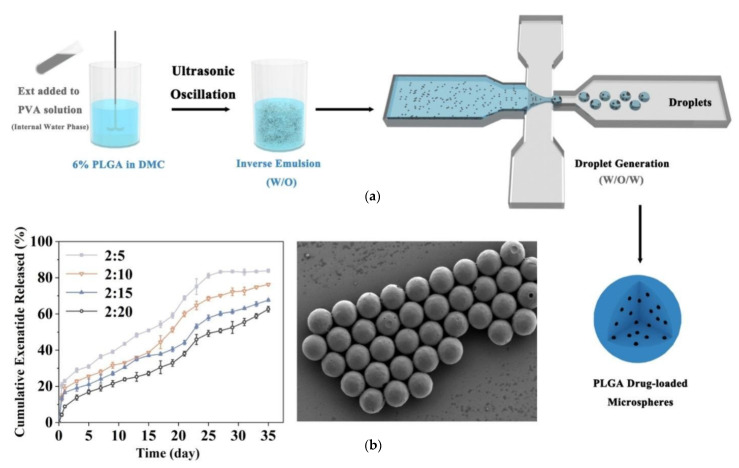
(**a**) The preparation of poly(lactic-*co*-glycolic acid) (PLGA) exenatide-loaded microspheres via water/oil/water (W/O/W) emulsion method with the use of microfluidic device. (**b**) The design of multi-channel droplet microfluidic devices [80].

The emulsification solvent evaporation method is a cornerstone for fabricating peptide-loaded PLGA particles, offering flexibility and scalability for various therapeutic applications. Variants like the double-emulsion (water–oil–water) technique are particularly advantageous for encapsulating hydrophilic peptides, such as MOG and plasmid DNA, as they protect the peptides during preparation and improve encapsulation efficiency [15,43,56]. This method’s adaptability extends to large-scale production using microfluidic flow-focusing systems, which ensure particle uniformity and scalability for drugs like exenatide (Figure 4) [80].

Optimizing process parameters, such as homogenization speed and solvent evaporation rate, allows for precise control over particle size, enhancing cellular uptake and distribution [1,9]. Burst release, a common challenge, can be minimized by incorporating stabilizers like polyvinyl alcohol (PVA) or hydrophilic agents like HP-beta-CD, which also support sustained release profiles [50,78]. Moreover, maintaining an alkaline pH in the inner aqueous phase reduces peptide degradation, preserving bioactivity [45]. Surface functionalization with targeting ligands, such as TAT peptides, enhances specificity and uptake for applications like brain delivery [3,107]. The integration of PLGA nanoparticles into hydrogels has also proven effective for achieving controlled and prolonged drug release, particularly for immune modulation applications [36,108]. However, challenges such as residual solvent removal and peptide acylation during PLGA degradation highlight the need for meticulous post-processing and stabilization [94].

### 4.2. Surface Functionalization and Advanced Coating Techniques

Surface functionalization plays an essential role in enhancing the bioactivity, specificity, and therapeutic potential of peptide-loaded PLGA systems. Functionalization strategies such as thiol–maleimide chemistry and maleimide–thiolether conjugation stabilize peptide attachment, enabling precise targeting and improved therapeutic outcomes [55,82]. Coatings like polydopamine have been shown to enhance cell adhesion and osteogenic activity, making them highly effective for bone regeneration [69]. Similarly, graphene oxide coatings improve mechanical strength and hydrophilicity, broadening the applications of PLGA in wound healing and tissue engineering [59]. Functionalization with cell-penetrating peptides (CPPs) such as transactivator of transcription (TAT) or erythrocyte membranes expands the potential for crossing biological barriers like the blood brain barrier [107,109]. These modifications not only increase cellular uptake but also enable targeted delivery, making them critical for applications in neurology, oncology, and tissue regeneration. By integrating advanced coating techniques, researchers can significantly enhance the therapeutic impact of PLGA-based systems across diverse applications.

### 4.3. Alternative Fabrication Techniques

Alternative methods like nanoprecipitation, electrospinning, and 3D printing offer unique advantages for specific applications, complementing traditional emulsion techniques. Nanoprecipitation, for example, provides an environmentally friendly, solvent-efficient approach ideal for hydrophobic drug delivery. This method is simple and rapid, requiring minimal solvent use, but may necessitate stabilizers or co-solvents to encapsulate hydrophilic peptides effectively [6,94,110]. Electrospinning, on the other hand, produces nanofibers functionalized with bioactive peptides such as RADA16 or BMP-2, offering a high surface-area-to-volume ratio and precise control over scaffold architecture, making it ideal for neural repair and wound healing [38,51]. Similarly, 3D printing allows for unparalleled control over the scaffold structure, incorporating peptides like BMP-9 to enhance mechanical properties and mineralization for bone defect repair [49,111]. These advanced fabrication techniques continue to expand the horizons of PLGA applications, offering tailored solutions for regenerative medicine and specialized drug delivery.

### 4.4. Guidelines for Optimizing Key Parameters

Optimizing preparation methods for peptide-loaded PLGA products is crucial for achieving the desired therapeutic outcomes. In emulsion-based systems, adjustments to solvent systems, stabilizer concentrations, and homogenization speeds ensure uniform particle size, high encapsulation efficiency, and controlled release profiles [1,9,78]. Surface coatings such as polydopamine or graphene oxide further enhance mechanical properties and biological interactions, particularly in tissue engineering [59,69]. Release kinetics can be refined through techniques like pH neutralization and the use of stabilizers, such as calcium phosphate salts, to inhibit peptide acylation and maintain bioactivity over extended periods [93,98,112]. Mechanistic modeling, including tri-phasic release analysis, allows for the precise tailoring of burst, erosion, and terminal phases to match specific therapeutic needs [22]. These optimization strategies enable researchers to align PLGA system design with the requirements of various applications, ensuring efficient and effective delivery.

### 4.5. Integration with Complementary Materials

**Figure 5:**
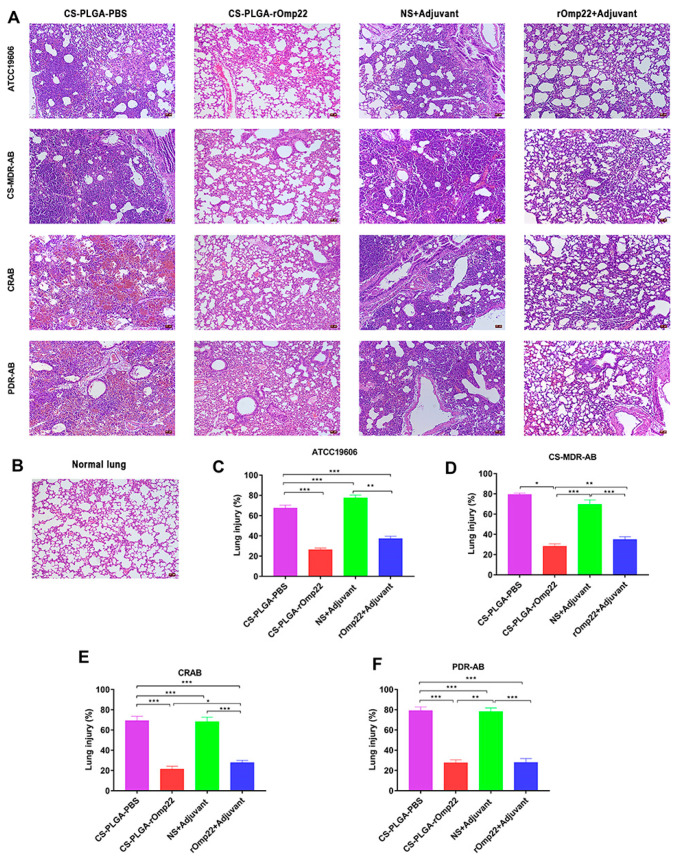
Histopathology of lung tissue specimens stained with haematoxylin-eosin and observed under a microscope (200×) taken from six mice with *A. baumannii* from each group at 24 h post-challenge. (**A**) Lung tissue from different immunized groups subjected to *A. baumannii* ATCC 19606 and three clinical *A. baumannii* strains (scale bar, 50 μm). (**B**) Lung tissue from unimmunized uninfected normal BALB/c mice with normal histological characteristics (scale bar, 50 μm). (**C**–**F**) Semiquantitative analysis of the inflammatory area in the lung tissue (n = 6). The histograms with mean percentage of lesion area within the total lung. Data are presented as the means ± SD (n = 6). * *p* < 0.05, ** *p* < 0.01, *** *p* < 0.001. Adopted with permission [30].

The integration of PLGA with complementary materials significantly enhances its functionality, broadening its application across diverse therapeutic areas. Chitosan/PLGA blends, for instance, extend release durations in vaccine formulations, particularly for combating multidrug-resistant pathogens (Figure 5) [30]. Composites like PLGA/nano-hydroxyapatite improve bioactivity and osteointegration, making them ideal for bone regeneration applications [60,92]. Similarly, ocular and pulmonary delivery systems benefit from composite designs that enhance drug retention and bioavailability, as seen in formulations incorporating agents like Licochalcone-A [76,108]. These integrations highlight the versatility of PLGA systems and their ability to address complex therapeutic challenges, paving the way for innovative solutions in drug delivery and regenerative medicine.

### 4.6. Optimization Strategies

Optimization strategies are critical for refining peptide-loaded PLGA systems to achieve maximum therapeutic efficacy. Techniques such as factorial design and the Box–Behnken Design (BBD) are used to optimize formulations for ocular and pulmonary delivery [108,110]. Simulated accelerated release methods allow for the prediction of long-term performance, facilitating the creation of customized release profiles for drugs like somatostatin analogs [13,22]. Adjusting osmotic pressure and encapsulation parameters further improves the therapeutic potential of peptides such as OH-CATH30 [17].

### 4.7. Stabilization Techniques

Ensuring effective stabilization is essential for maintaining peptide functionality within PLGA formulations. Approaches like incorporating carboxymethyl chitosan and inorganic cations reduce peptide acylation, preserving stability during degradation [44,94]. Additionally, freeze-drying and nebulization techniques maintain bioactivity and enable efficient pulmonary delivery for respiratory therapies [113]. Intrinsic porogens are often employed to optimize release kinetics, ensuring consistent therapeutic effects [44].

Table 3 highlights the variety of methodologies used in preparing peptide-loaded PLGA systems, from traditional emulsion techniques to advanced 3D printing and surface functionalization. Each method is aligned with specific therapeutic goals, ensuring precision in encapsulation, stability, and delivery. Patterns emphasize how innovations like nanoprecipitation, hydrophobic ion pairing, and functionalized scaffolds enhance encapsulation efficiency, control release, and optimize bioactivity. These methodologies reflect a balance between scalable production and tailored functionality, supporting applications in cancer therapy, tissue regeneration, and immunomodulation.

## 5. Key Physicochemical Features in PLGA Nanocarriers

Peptide-loaded PLGA systems possess a range of physicochemical properties that make them highly adaptable for various medical applications. Particle size is a critical factor, varying from 27 nm to 558 nm for nanoparticles, which are used for targeted drug delivery in conditions such as Alzheimer’s disease and bacterial infections [2,82,103]. Larger structures, such as microspheres designed for Triptorelin acetate, reach sizes up to 35.3 µm, enabling sustained drug release over extended periods [78]. Advanced designs, such as grooved PLGA films with 800 nm ridges, enhance cellular alignment and are particularly suitable for skeletal muscle engineering [88].

### 5.1. Surface Stability and Charge

The surface charge and stability of PLGA nanocarriers significantly influence their bioactivity and interaction with biological systems. Zeta potentials for these systems range from −46.0 mV to +1.65 mV, depending on surface modifications [9,110]. Specific coatings, such as chitosan (+4.39 mV) and Pep5 modifications (+20.01 mV), enhance colloidal stability and cellular uptake, which are crucial for effective drug delivery in cancer therapies and wound-healing applications [30,119].

### 5.2. Encapsulation Efficiency and Release Profiles

Encapsulation efficiency in peptide-loaded PLGA products is consistently high, typically exceeding 70%, and reaching as much as 96.56% in systems like BAMLET and octreotide acetate [93,120]. These systems offer tailored release profiles to meet diverse therapeutic requirements, from rapid initial release to sustained delivery over extended periods. For example, BMP-2 systems retain their activity for weeks, supporting bone regeneration [19], while self-immolative-protecting groups ensure a controlled release for up to 50 days [98].

### 5.3. Biocompatibility and Bioactivity

PLGA systems are highly biocompatible and bioactive, making them suitable for a variety of medical applications. For instance, systems designed for insulin delivery and oral vaccines demonstrate non-cytotoxicity and safety [75,102]. Poly(dopamine)-coated PLGA scaffolds improve osteogenic differentiation and tissue integration, proving effective in bone regeneration [92]. Additionally, RADA16-I-BMHP1 nanofibers facilitate Schwann cell proliferation and gene expression, making them instrumental in neural repair [51,52].

### 5.4. Therapeutic Efficacy Across Applications

**Figure 6:**
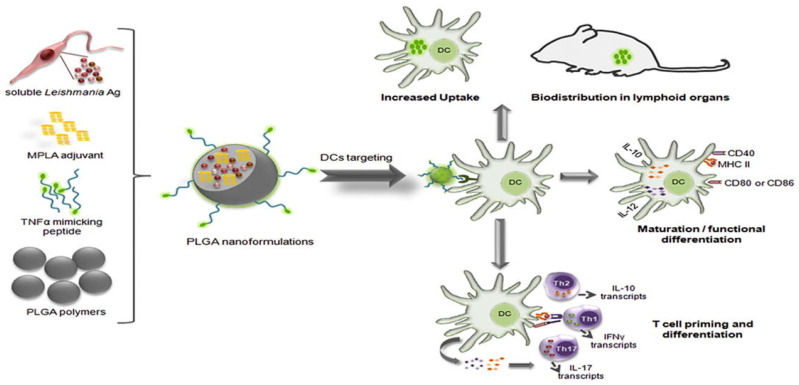
PLGA NPs surface-modified with a TNF-α-mimicking peptide encapsulated with soluble *Leishmania* antigens (sLiAg) and MPLA adjuvant. The synthesized PLGA NPs exhibited low cytotoxicity levels, efficient uptake by dendritic cells (DCs), induced maturation and functional differentiation, and increased stimulation of IL-12 and IL-10 production. Adopted with permission [121].

The therapeutic efficacy of peptide-loaded PLGA systems spans a wide range of medical fields. Functionalized nanoparticles, such as those incorporating TNF-alpha-mimicking peptides, activate dendritic cells and prime T-cells, demonstrating promise for vaccine applications (Figure 6) [121]. In Alzheimer’s therapy, BSBP8 nanoparticles reduce amyloid beta levels by 82%, significantly improving cognitive function [27]. For antimicrobial applications, peptides like MSI-78(4-20) and KSL-W provide sustained antibacterial activity and effectively disrupt biofilms, addressing infections such as periodontitis [55,64].

### 5.5. Advanced Applications and Complex Challenges

PLGA nanocarriers are increasingly utilized in advanced and complex medical applications. BMP-9 and GFOGER peptide-functionalized PLGA scaffolds have shown significant improvements in bone density and mineralization, making them highly effective in bone repair [111]. Graphene oxide and RGD peptide-functionalized fibers support tissue regeneration, particularly in promoting smooth muscle and myoblast differentiation [103]. Cutting-edge designs, such as curcumin/cisplatin nanoparticles and BMP-2-loaded fibers, have demonstrated precise targeting capabilities, achieving superior outcomes in glioblastoma treatment and chronic wound healing [37,109].

Table 4 describes the physicochemical properties critical to the design and performance of peptide-loaded PLGA systems, including size, surface charge, and encapsulation efficiency. Patterns indicate that precise size control enhances tissue penetration, while surface modifications optimize cellular uptake and colloidal stability. Tri-phasic release profiles align with therapeutic needs for chronic conditions. The data highlight the customization potential of PLGA systems in tailoring bioactivity, release profiles, and structural compatibility for applications like bone regeneration, cancer therapy, and neuroprotection.

## 6. Evaluation Metrics and Methods for Peptide-Loaded PLGA Nanocarriers

Peptide-loaded PLGA systems undergo thorough evaluation to ensure their safety, efficacy, and suitability for a wide array of therapeutic applications. These evaluations focus on physicochemical properties, structural integrity, biological interactions, and in vivo performance.

### 6.1. Physicochemical and Structural Characterization

Characterizing the physicochemical properties of peptide-loaded PLGA systems is the first step in their development. Analytical techniques such as scanning electron microscopy (SEM), atomic force microscopy (AFM), and transmission electron microscopy (TEM) are employed to assess particle size, zeta potential, and morphology [1,36,57,124,125].

Encapsulation efficiency and stability are evaluated using methods like ALP assays, MALDI-TOF-MS, and high-performance liquid chromatography (HPLC) to confirm peptide bioactivity [94,95,98]. Material interactions and thermal stability are further investigated using spectroscopic techniques (FTIR, NMR) and circular dichroism [27,93,116]. Advanced functionalization methods, including polydopamine-assisted modifications, and fabrication techniques like electrospinning facilitate the creation of innovative material designs [91,111,114].

### 6.2. Controlled Release Profiles

The ability to control the release of therapeutic peptides is crucial to their effectiveness. In vitro studies evaluate burst and sustained release profiles under varying conditions, including pH [11,14,108,124]. For instance, BMP-2-loaded PLGA systems deliver peptides slowly, enhancing osteoinductive properties in bone regeneration [19,115]. Techniques such as mathematical modeling and flow-through systems are used to optimize polymer degradation and release kinetics, ensuring consistency [14,42,112].

### 6.3. Cellular and Molecular Bioactivity

The cellular uptake and bioactivity of PLGA nanoparticles are explored through mechanisms like macropinocytosis and endosomal escape, which optimize peptide delivery [29,57,118]. Additional studies, including immune activation assays and cytokine production analyses, validate their suitability for vaccine applications [33,45,126]. In regenerative medicine, osteoblast development, cellular differentiation, and apoptosis induction are monitored to evaluate therapeutic potential [6,50,92].

### 6.4. Immune-Modulatory Properties

Peptide-loaded PLGA formulations play a critical role in immunotherapy and vaccine development. Evaluations include T-cell response assays, cytokine profiling, and tests for antigen-specific immune activation [32,121,127]. Studies such as immunogenic cell death assays and PD-L1 blockade experiments assess their potential in cancer immunotherapy [46]. Computational and experimental epitope design methodologies further refine vaccine candidates [32,126].

### 6.5. Therapeutic Efficacy and Safety

**Figure 7:**
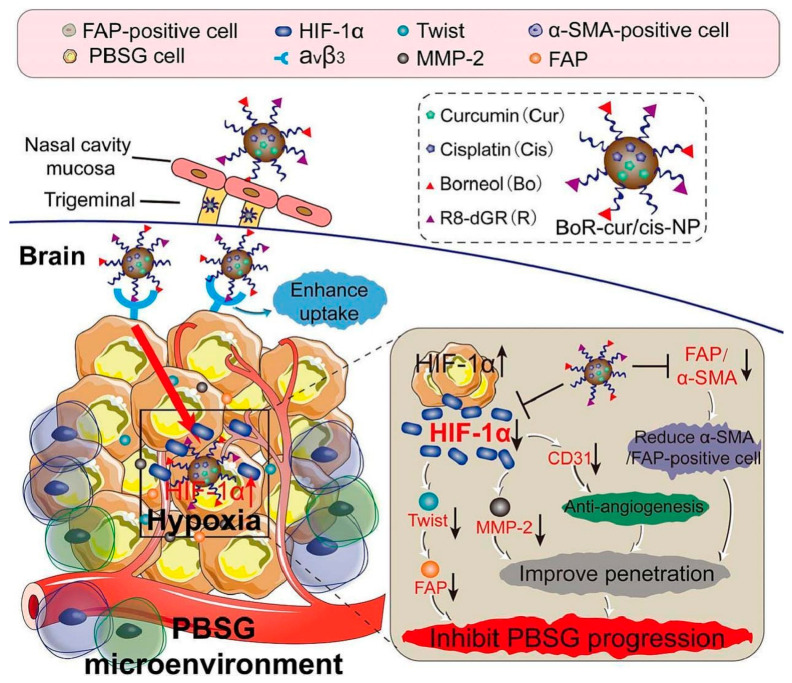
Intranasal administration of borneol (Bo)/R8dGR peptide-modified PLGA-based nanoparticles (NP) co-loaded with curcumin and cisplatin (cur/cis). This nano-formulation improved the brain penetration via reduction of the expression of ZO-1 and occluding in nasal mucosa, while the R8dGR peptide modification improved the targeting of the NP by binding to integrin αvβ3 receptors and reduced hypoxia and angiogenesis in the PBSG microenvironment. Adopted with permission [109].

Therapeutic efficacy and safety are validated through extensive in vitro and in vivo testing. Tumor-targeting capabilities are assessed via tumor retention, survival analyses, and cancer cell inhibition assays [47,109,125]. For instance, Figure 7 demonstrates the intranasal delivery of Borneol/R8dGR peptide-modified PLGA nanoparticles co-loaded with curcumin and cisplatin for pediatric brainstem glioma treatment [109].

### 6.6. Applications in Regenerative Medicine

In regenerative medicine, evaluations include testing for bone and cartilage repair using 3D-printed scaffolds, monitoring osteogenic marker expression, and conducting in vivo mineralization studies [38,50,111]. Skin regeneration studies focus on epithelialization, angiogenesis, and the formation of granulation tissue [59,97]. Similarly, applications in nerve and muscle repair involve neurite alignment studies, Schwann cell behavior analysis, and myoblast differentiation assessments [52,88,103]. Neurotherapeutic applications include assessments of cognitive improvements and biochemical markers in Alzheimer’s disease models [5,8,48].

### 6.7. Antimicrobial Properties

Antimicrobial properties are demonstrated through tests for biofilm inhibition, reductions in bacterial burden, and antimicrobial peptide activity [21,55,128]. Sustained antifungal efficacy is also evaluated, including effects against Cryptococcus neoformans, showing potential for managing infections and promoting wound healing [20,30,123].

### 6.8. Advanced Functional Studies

Advanced studies investigate targeted delivery and tumor penetration. For example, integrin receptor targeting and hypoxia mitigation are evaluated for improved tumor-specific delivery [109,125]. Apoptosis pathways are analyzed using methods such as caspase-3 staining and mitochondrial function assays [7,119]. Neurotherapeutic studies address challenges like blood brain barrier (BBB) targeting and amyloid plaque reduction for Alzheimer’s disease [8,27,48].

### 6.9. Process Optimization

Scalable production methods are a key focus. Comparisons between microfluidic techniques and bulk production methods help evaluate efficiency [41,80]. Quality by Design (QbD) methodologies optimize critical process parameters, ensuring reproducibility and performance [42,78]. Statistical tools such as response surface methodology and precision testing further enhance formulation reliability [22,90,110].

Table 5 outlines the comprehensive evaluation metrics used to validate the safety, efficacy, and performance of peptide-loaded PLGA systems. These include physicochemical characterization, release kinetics, and in vivo assessments of biocompatibility and therapeutic efficacy. Patterns emphasize the integration of multidisciplinary testing, such as imaging techniques, immune response assays, and mechanical property evaluations. The table showcases a rigorous approach to ensuring product stability, optimized release, and targeted delivery, highlighting their potential in advancing therapies for cancer, tissue regeneration, and infectious diseases.

## 7. Milestones and Multifunctional Capabilities of PLGA Nanocarriers

Peptide-loaded PLGA systems represent a significant advancement in sustained drug delivery, effectively reducing dosing frequency while enhancing therapeutic outcomes. These nanocarriers demonstrate long-term efficacy across a wide range of applications, including bone regeneration, infection treatment, and the management of chronic systemic diseases. For instance, prolonged drug retention has been observed in ischemic injury and Alzheimer’s disease models, highlighting their potential for addressing complex medical challenges [3,9,36,63,77,83]. Functionalized PLGA nanoparticles enhance targeting and selectivity, as seen with T7 peptide-functionalized micelles for glioblastoma and renal-targeting peptides for kidney-specific delivery, both of which improve therapeutic precision [2,25,61,62].

### 7.1. Versatile Applications

The versatility of peptide-loaded PLGA systems spans neural repair, autoimmune disease management, and infectious disease prevention. Their applications range from glioblastoma therapies to nanovaccines for zoonotic infections, demonstrating their broad applicability and effectiveness [15,30,53,56,104]. Targeted delivery minimizes systemic toxicity and enhances bioavailability, as observed in therapies like radionuclide treatment and thrombolysis, which reduce side effects such as renal toxicity and bleeding risk [12,16,132].

### 7.2. Innovative Delivery Platforms

Cutting-edge delivery platforms, such as composite hydrogels, supramolecular nanofibers, and hybrid scaffolds, are transforming drug delivery and tissue regeneration. These advanced materials improve drug retention and bioavailability in applications like mucosal delivery and bone repair [36,92,113]. Additionally, PLGA systems have become integral to vaccine development, enabling prolonged antigen release and eliciting robust immune responses in vaccines targeting multidrug-resistant infections, influenza, and cancer [24,30,31,66].

### 7.3. Clinical Translation

Peptide-loaded PLGA systems are progressing toward clinical translation, supported by predictive IVIVC models and successful outcomes in treatments for Alzheimer’s disease and glioblastoma. These advances underscore the systems’ readiness for broader therapeutic adoption [1,25,39]. Enhanced intracellular and endosomal delivery capabilities, facilitated by cell-penetrating peptide-functionalized nanoparticles, ensure effective gene delivery and sustained expression in pulmonary therapies [26,56,57].

### 7.4. Controlled Release and Antimicrobial Properties

Controlled release mechanisms ensure long-term effects while minimizing drug instability. Examples include tri-phasic release profiles for somatostatin analogs and steady peptide delivery in antifungal therapies, addressing diverse therapeutic needs [13,22,116,123]. The antimicrobial capabilities of these systems are particularly notable, with formulations targeting MRSA, bacterial keratitis, and periodontitis, as well as photothermal therapy providing synergistic effects [17,20,21,64].

### 7.5. Biocompatibility and Safety

The consistent demonstration of biocompatibility and safety makes peptide-loaded PLGA systems suitable for managing chronic diseases, preventing allergies, and advancing tissue regeneration [70,75,129]. In neurotherapeutics, these systems effectively cross the blood brain barrier, reducing amyloid-beta aggregation in Alzheimer’s disease therapies and suppressing tumor growth in glioblastoma [4,27,116].

### 7.6. Advances in Vaccine Development

PLGA-based platforms play a pivotal role in vaccine development, offering enhanced immune responses, multi-epitope formulations, and extended antigen release. Applications include cross-reactive influenza vaccines and mucosal immunity for diseases like swine dysentery, showcasing their impact on global health challenges [31,87,121].

### 7.7. Tissue Engineering and Regenerative Medicine

In regenerative medicine, peptide-functionalized PLGA scaffolds demonstrate significant potential. Functional peptides such as BMP-2 and RADA16-I improve outcomes in bone, cartilage, and nerve repair, as well as promote angiogenesis in ischemic models [18,60,91,114].

### 7.8. Cancer Therapy Innovations

Innovations in cancer therapy have led to improved outcomes by enhancing tumor-specific delivery while reducing systemic toxicity. For example, SP94-functionalized nanoparticles for hepatocellular carcinoma achieve selective tumor targeting and high therapeutic efficacy [34,46,119].

### 7.9. Scalable Manufacturing and Quality Control

Efficient manufacturing techniques, including microfluidics and co-axial electrospraying, enhance scalability and reproducibility. These approaches are complemented by accelerated in vitro release testing, which ensures robust quality control and supports widespread application [13,42,80].

Table 6 summarizes the proven benefits of peptide-loaded PLGA systems, including sustained drug release, enhanced stability, and multifunctionality. These systems address critical therapeutic challenges like peptide instability, inefficient targeting, and dosing frequency. Patterns emphasize their versatility across applications such as neurotherapeutics, oncology, and vaccine delivery. The table underscores PLGA’s role in enabling advanced diagnostics, immune response activation, and tissue engineering, paving the way for scalable, personalized medicine solutions.

## 8. Overcoming Developmental and Translational Challenges in Peptide-Loaded PLGA Nanocarriers

Peptide-loaded PLGA systems face significant challenges in formulation design and stability. Peptide acylation and degradation in acidic microenvironments are persistent issues, with positively charged peptides posing additional challenges due to strong interactions with the negatively charged PLGA. Strategies such as calcium phosphate depots, hydrophobic ion-pairing, self-immolative-protecting groups, and dynamic surface coatings have shown promise, but universal solutions are still required to ensure stability across diverse physiological conditions [11,22,40,77,93,98,112,116].

To provide a comprehensive overview of these challenges, Table 7 summarizes the key applications of PLGA-based systems, highlighting their advantages and associated challenges across diverse fields such as cancer therapy, vaccine development, and tissue engineering. Insights include the potential of PLGA systems for targeted delivery, improved stability, and sustained release while addressing hurdles like scalability, cost, and variability in therapeutic outcomes. The table provides a comprehensive overview to guide strategic improvements in the formulation, functionalization, and clinical translation of PLGA-based technologies.

### Manufacturing Scalability, Cost, and Regulatory Barriers: Implications for Clinical Use

The challenges associated with manufacturing scalability, cost, and regulatory hurdles critically impact the clinical translation of peptide-loaded PLGA nanocarriers. Large-scale production requires high reproducibility, cost-effectiveness, and compliance with stringent regulatory standards, which are often difficult to achieve with current methodologies.

Manufacturing Scalability: Techniques such as 3D printing, electrospinning, and microfluidic technologies, while promising in controlled laboratory settings, struggle with scalability for industrial production [37,40,41,51,69,80,86,91]. The precision required for incorporating multifunctional elements like targeting ligands or dual-drug systems complicates automation and increases production time. For example, microfluidic methods require costly modifications to achieve the throughput needed for large-scale applications, which hinders the transition from laboratory prototypes to market-ready products.

Cost Constraints: Advanced materials and processes used in peptide-loaded PLGA systems, such as PEGylation, gold nanoparticle incorporation, and hydrophobic ion-pairing, significantly increase production costs [3,20,59,108,115]. While these components enhance therapeutic outcomes, their high costs make large-scale production economically prohibitive. Furthermore, resource-intensive strategies, such as T-cell membrane-coated nanoparticles, further inflate costs, limiting accessibility and affordability for widespread clinical use.

Regulatory Barriers: The regulatory requirements for complex peptide-loaded PLGA systems present significant obstacles [7,8,27,28,38,47,109,118]. Each added functionality, such as targeting ligands or dual-drug formulations, necessitates rigorous safety and efficacy evaluations. Regulatory approval processes demand consistent product quality and compliance with Good Manufacturing Practice (GMP) standards. However, the inherent variability in PLGA systems due to batch-to-batch differences in polymer characteristics complicates reproducibility and standardization, further slowing clinical translation.

Clinical Impact: These challenges collectively limit the deployment of peptide-loaded PLGA nanocarriers in clinical settings. Despite demonstrating significant promise in preclinical studies, such as sustained drug release and enhanced targeting for cancer therapy and neurodegenerative diseases, many systems fail to transition to clinical trials [3,27,62,74,106,107]. The inability to achieve large-scale, cost-effective production undermines the feasibility of widespread application, while regulatory delays and high costs reduce the commercial appeal of these technologies.

## 9. Next Generation of Peptide-Loaded PLGA Systems

Future developments for peptide-loaded PLGA systems focus on enhancing customization and scalability, addressing challenges in mass production while maintaining precision. Techniques such as 3D printing and microfluidics must evolve to support the synthesis of complex systems, including dual-drug hydrogels and composite scaffolds. Incorporating Quality by Design (QbD) principles will help ensure consistent production and regulatory compliance, making these systems more accessible [42,69,80,100].

Integrating multifunctional systems capable of therapeutic, diagnostic, and regenerative applications is a key direction. These platforms will combine properties such as antimicrobial, osteogenic, and angiogenic functions, which are critical for wound healing and tissue engineering. Stimuli-responsive components, such as light-activated nanoparticles, offer promising applications in precision oncology [23,68,91,125].

### 9.1. Patient-Specific Applications and Personalized Medicine

Emerging trends in personalized medicine highlight the need for peptide-loaded PLGA systems tailored to individual patient profiles. These systems can be customized to align with specific disease pathologies, genetic markers, or immune responses. For example, patient-derived biomolecules could be integrated into PLGA scaffolds, ensuring compatibility and enhancing therapeutic outcomes in tissue engineering [25,51,59,91,111].

In oncology, personalized nanoparticles targeting specific tumor markers, such as HER2-positive breast cancers or EGFR-overexpressing glioblastomas, demonstrate potential for precision drug delivery. Ligand-functionalized PLGA systems tailored to these markers can improve therapeutic outcomes and reduce off-target effects [28,34,61,84]. Similarly, for neurodegenerative disorders like Alzheimer’s and Parkinson’s, tailored brain-targeting platforms incorporating disease-specific ligands could enhance drug distribution across the blood/brain barrier, accommodating variability across patient populations [29,106,129].

Personalized vaccine platforms also represent a transformative application of peptide-loaded PLGA systems. Multi-epitope nanoparticle systems, developed using tumor-specific neoantigens, offer the potential for individualized cancer immunotherapies. Self-adjuvant designs enhance immune activation, providing significant benefits in vaccine efficacy for infectious diseases and emerging zoonotic pathogens [7,30,31,32,118].

In regenerative medicine, patient-specific bioengineered scaffolds incorporating individualized growth factors or extracellular matrix components could offer customized solutions for complex tissue defects. Applications include scaffolds for bone regeneration infused with patient-specific osteoinductive molecules or neural repair systems optimized for the localized delivery of neuroprotective peptides [19,91,111].

### 9.2. Enhancing Precision and Stability

Advancements in targeting mechanisms and peptide stabilization techniques further support patient-specific applications. Ligands designed to target renal, endothelial, or bone tissues can be tailored to the unique physiological conditions of individual patients, enhancing precision and therapeutic efficacy [29,34,61,84]. Stabilization strategies, including hydrophobic ion-pairing and calcium phosphate depots, address the variability in peptide degradation and clearance, ensuring consistent therapeutic effects across diverse populations [11,22,93,98].

### 9.3. Clinical Translation and Future Directions

Transitioning these technologies to clinical practice will require extensive validation in patient-specific contexts. Clinical trials targeting diseases such as glioblastoma and Alzheimer’s, as well as chronic wounds must incorporate diverse patient populations to evaluate efficacy and safety comprehensively [5,25,27,115,125]. For example, personalized treatments for glioblastoma can leverage tumor-specific biomarkers to refine targeting and improve therapeutic responses.

Incorporating artificial intelligence and machine learning may further advance patient-specific applications by optimizing nanoparticle design and predicting release profiles based on individual patient data. These innovations will enable peptide-loaded PLGA systems to align with the growing emphasis on precision medicine, offering highly tailored therapeutic solutions [2,86,118].

## 10. Conclusions

Peptide-loaded PLGA nanocarriers have emerged as pivotal tools in addressing the challenges associated with peptide therapeutics, offering transformative potential in various biomedical applications. Their inherent biodegradability, biocompatibility, and capacity for controlled and targeted peptide delivery make them highly effective for addressing therapeutic needs in areas such as cancer treatment, neurodegenerative disorders, and infectious diseases. Advanced surface functionalization and hybrid polymer designs have further expanded their applications to regenerative medicine and vaccine development, enhancing immune responses and promoting tissue repair.

Despite these advances, significant hurdles remain, particularly in scalability, cost-effectiveness, and achieving regulatory approval. Future research should focus on specific areas within polymer chemistry to address these challenges effectively. For instance, the development of novel copolymer systems could improve the mechanical stability, degradation rates, and drug-release profiles of PLGA carriers, enabling more reliable therapeutic delivery. Refining surface modification strategies, such as grafting ligands or functional groups onto PLGA surfaces, may enhance cellular targeting and bioavailability, while responsive polymer chemistry could facilitate site-specific peptide release through stimuli-responsive designs. Additionally, investigating advanced polymer architectures, such as block polymers or star-shaped polymers, could further enhance control over degradation and release kinetics. Emphasis on green chemistry approaches in polymer synthesis would also align innovation with sustainability goals, addressing environmental concerns during material development.

In a clinical context, these advancements could directly support the development of cancer immunotherapies, peptide-based treatments for neurodegenerative conditions, and targeted vaccine platforms for infectious diseases. Collaboration across disciplines, including nanotechnology, pharmaceutical engineering, and clinical sciences, will be essential to optimize production processes, enhance scalability, and expedite regulatory pathways. By addressing these focused areas, peptide-loaded PLGA nanocarriers hold the potential to drive significant progress in therapeutic delivery and play a central role in the evolution of personalized medicine.

## Figures and Tables

**Table 1:** Therapeutic applications and functional enhancements of peptide-loaded PLGA systems.

Therapeutic Area	Key Peptides and Active Ingredients	Therapeutic Purpose	Clinical Significance and Addressed Limitations	References
Antimicrobial and Anti-Infective	SAAP-148, OP-145, LL37, OH-CATH30, G17/G19, KSL-W, Dermaseptin-PP	AMR bacteria, biofilm inhibition, wound healing, bone infection treatments.	Tackles antibiotic resistance and biofilm persistence with enhanced localized delivery and extended antibacterial activity.	[9,17,20,21,23,63,64]
Vaccines and Immunotherapy	ESAT-6, Zika virus peptide, influenza peptides, NY-ESO-1	Vaccines for TB, Zika, influenza A, Toxoplasma gondii, and cancer.	Stable, controlled-release vaccine platforms address diseases with limited existing prophylactic options.	[24,31,32,65,66]
Cancer Therapy	Vincristine, doxorubicin, IMM60, TAT, SP94	Targeted therapies for breast, prostate, melanoma, hepatocellular glioblastoma, neuroendocrine cancers.	Tumor specificity, reduced systemic toxicity, improved outcomes in aggressive cancers.	[2,6,34,39,47]
Cancer Therapy	Multi-epitope peptides, STEAP1	Cancer vaccines and immunotherapy.	Robust immune responses and improved antigen-specific T-cell activation for advanced cancer treatment.	[33,66,67]
Neurological Disorders	Carmustine, curcumin, Tet-1 peptide, NAP peptide	Alzheimer’s, glioblastoma, neurodegenerative diseases.	Overcomes BBB penetration challenges, poor bioavailability, and limited efficacy of traditional therapies.	[3,4,5,8,25,68]
Bone and Tissue Engineering	BMP-2, P24, Teriparatide, Fusion peptides	Bone regeneration, osteogenesis, tissue repair.	Addresses slow healing and infection risks in orthopedic applications with multifunctional scaffolds and sustained release.	[18,19,36,38,60,69]
Autoimmune and Allergy Therapies	IL-10, IMM60, CpG ODN, MOG peptide, BLG-Pep	Autoimmune disease management, allergy prevention, and immune modulation.	Antigen-specific tolerance and long-term immune regulation to combat chronic inflammation and allergies.	[15,32,66,70,71,72]
Gene and RNA Therapies	Plasmid DNA, siRNA, antisense oligonucleotides	Gene therapy for lung diseases, obesity, cancer.	Effective intracellular delivery and endosomal escape address challenges of systemic nucleic acid therapies.	[56,57,73,74]
Anti-inflammatory Therapies	Fluorometholone, Asiatic acid	Ocular and kidney inflammatory disorders.	Provides safe, targeted anti-inflammatory effects, reducing systemic side effects.	[61,75,76]
Hormonal Peptides	Leuprolide, goserelin, octreotide	Sustained release for hormone-related conditions like prostate cancer and fertility.	Overcomes short half-life and frequent dosing requirements with long-acting formulations.	[1,44,77,78]
Radiolabeled Agents	Lutetium-177 DOTATATE	PRRT for neuroendocrine tumors.	Reduced renal radiation dose with high therapeutic efficacy using PEG-coated formulations.	[12]
Diabetes Therapies	Insulin, GLP-1 analogs	Oral and intranasal delivery for diabetes management.	Overcomes peptide stability and bioavailability issues in oral and nasal formulations.	[29,79,80]
Wound Healing and Regeneration	L-carnitine-GHK, LL37, BMP-2	Chronic wound management and tissue repair.	Promotes neovascularization, collagen deposition, and faster healing in hard-to-treat wounds.	[19,23,59,81]
Cardiovascular Agents	Imatinib, WKYMVm	Atherosclerosis and ischemia treatments.	Improves endothelial function and promotes angiogenesis to address poor vascular regeneration.	[82,83,84]
Antiviral Therapies	Oseltamivir, HIV-TAT peptide	COVID-19, HIV, and viral therapies.	Targeted delivery and sustained antiviral activity reduce frequent dosing requirements.	[54,68]
Antibacterial Peptides	KSL-W, Dermaseptin-PP	Oral infections and antibacterial wound therapy.	Combines sustained antimicrobial action with synergistic effects for resistant bacterial infections.	[20,64]
Alzheimer’s and Neuroprotection	Curcumin, NAP peptide, amyloid inhibitors	Neuroprotection, amyloid reduction, and memory enhancement.	Targets amyloid aggregation and oxidative stress, key challenges in Alzheimer’s disease management.	[3,4,5,8,27]

**Table 2:** PLGA compositions and their roles in PLGA-based systems.

Polymer/Excipient Composition	Applications (Pattern Observed)	Preparation/Processing Highlights	Significance in Clinical Outcomes	Reference
PLGA with PEG coatings	Cancer therapies, brain targeting, ocular delivery. Enhanced circulation, bioavailability, and targeting specificity.	Emulsion solvent evaporation with PEG conjugation for prolonged circulation.	PEG enhances water solubility and prevents opsonization, allowing for prolonged circulation time and improved drug delivery to specific tissues. Clinically, this improves therapeutic efficacy and minimizes systemic side effects.	[4,9,12,25,34,75,76]
Acid- and ester-terminated PLGA	Controlled release systems for positively charged peptides. Reduced acylation and optimized release kinetics.	Emulsion solvent evaporation, hydrophobic ion pairing, pH-controlled environments.	Termination chemistry allows for fine-tuning drug release profiles and reduction of peptide degradation. Clinically, this ensures more consistent therapeutic outcomes and reduces the frequency of dosing.	[10,11,43,77,93,98]
PLGA with chitosan or chitosan-based blends	Vaccine delivery (oral/nasal), mucoadhesive systems, antibacterial applications.	Chitosan provides enhanced mucoadhesion and immune response stimulation.	Chitosan increases mucoadhesion, improves residence time at mucosal surfaces, and stimulates immunogenicity for vaccines. Clinically, this enables more effective vaccine delivery and localized antibacterial effects.	[30,64,94,100,101]
PLGA with cell-penetrating peptides (CPPs)	Intracellular delivery, brain therapies, gene delivery, and Alzheimer’s treatment.	Double-emulsion techniques and surface modifications for enhanced uptake and intracellular targeting.	CPPs facilitate cellular uptake, enabling efficient delivery of therapeutics to intracellular targets. This is crucial for diseases like Alzheimer’s, where intracellular pathways are involved.	[25,26,29,56,102]
PLGA with gold nanoparticles/graphene oxide	Neural regeneration, tissue engineering, antimicrobial applications. Conductivity and biocompatibility enhance therapeutic efficacy.	Electrospinning, nanoparticle surface modification, and 3D printing.	The addition of conductive materials like gold or graphene oxide promotes cell signaling and tissue regeneration while maintaining antimicrobial properties. Clinically, this enhances the success of tissue engineering and wound healing.	[59,85,103,104]
PLGA with bone-targeting peptides (e.g., BMP-2, P24)	Bone regeneration, osteogenic differentiation, and skeletal engineering.	Incorporation into 3D scaffolds, hydrogels, or composites via emulsion techniques.	Bone-targeting peptides enhance osteogenic activity, leading to faster and more effective bone repair and regeneration. Clinically, this is pivotal in treating fractures and bone disorders.	[18,19,36,38,69,105]
PLGA-encapsulating antigenic peptides and vaccines	Immunotherapy, cross-protective vaccine platforms, cancer vaccines.	Double-emulsion and nanoprecipitation for antigen stability and immune response optimization.	Encapsulation stabilizes antigenic peptides, enhancing immune response and enabling precise targeting of tumors or pathogens. Clinically, this improves vaccine effectiveness and reduces the need for boosters.	[24,31,32,33,65]
PLGA with antimicrobial peptides	Infection control, wound healing, and bacterial biofilm inhibition.	Sustained release microspheres with synergistic photothermal effects.	Sustained release of antimicrobial peptides ensures continuous protection against infections, making it valuable for wound healing and chronic infection management.	[17,20,55,59,63]
PLGA for Alzheimer’s and brain disorders	Enhanced blood/brain barrier penetration, neuroprotection, and memory improvement.	Functionalized nanoparticles with BBB-targeting peptides.	Functionalization improves blood/brain barrier crossing, delivering neuroprotective drugs directly to the brain. Clinically, this offers targeted treatment for neurodegenerative disorders like Alzheimer’s.	[3,4,5,8]
PLGA with hybrid polymer blends (e.g., PEG, polycaprolactone)	Sustained release systems for dual-drug delivery in cancer therapy and tissue regeneration.	Nanoprecipitation and emulsion blending with additional polymers.	Hybrid blends enable the co-delivery of multiple drugs with distinct release profiles, enhancing therapeutic efficacy in complex diseases like cancer.	[34,46,74,106]
PLGA modified with T-cell receptors or peptides	Enhanced targeting and retention in cancer therapies.	Functionalized PLGA with receptor-specific peptides for precision delivery.	Targeting T-cell receptors improves immune response precision, reducing off-target effects and enhancing cancer immunotherapy outcomes.	[6,22,47,62]
PLGA combined with stabilizers or hydrogels	Prolonged stability and bioactivity of peptides for bone and vascular regeneration.	Microspheres integrated into hydrogels with stabilizers like HP-β-CD and BSA.	Stabilizers and hydrogels extend drug stability and improve bioactivity, ensuring sustained therapeutic effects in bone and vascular repair.	[19,38,95]

**Table 3:** Methodologies to prepare peptide-loaded PLGA products.

Preparation/Processing Method	Key Patterns	Enhanced Observations	Reference
Double-emulsion Solvent Evaporation	Encapsulation of peptides, proteins, and antigens with controlled release and reduced burst release.	Achieved high encapsulation efficiency for immunogenic antigens, stable vaccine delivery, and long-term peptide release.	[1,13,15,19,32,63]
Emulsion Solvent Evaporation	Hydrophobic drug encapsulation, functionalization, and vaccine formulations.	Supported safe delivery of antimicrobial peptides, vaccines, and anticancer agents with sustained release profiles.	[9,12,24,32,83,93]
Nanoprecipitation Method	Narrow size distribution and high stability for small drugs and peptides.	Optimized using Box–Behnken and similar designs for enhanced targeting and biocompatibility.	[74,80,106,110]
Electrospinning	Scaffolds for tissue engineering applications, especially neural and bone regeneration.	Promoted neuronal differentiation and angiogenesis with bioactive peptide incorporation in hybrid scaffolds.	[52,85,88,104,114]
Co-encapsulation of Peptides/Drugs	Multi-drug delivery for synergistic release of peptides and chemotherapeutics.	Enhanced efficacy of cancer therapies by co-encapsulating immune-stimulating peptides with chemotherapeutics.	[66,94,115]
PEGylation and Surface Functionalization	Improved biocompatibility, stability, and specific targeting through functionalization.	Enabled tumor targeting, BBB crossing, and intracellular uptake for advanced drug delivery systems.	[6,25,26,35,39]
Hydrophobic Ion Pairing	Stabilizes hydrophilic peptides, enhancing encapsulation and release control.	Ion pairing reduced burst release, improved peptide loading, and maintained drug integrity over extended periods.	[11,40,112]
3D Printing of Scaffolds	Allows for precise architecture for bone and tissue regeneration with bioactive modifications.	Supported controlled BMP-2 and peptide release for synergistic osteogenic and antibacterial effects.	[36,38,60,91,111]
Freeze-Drying (Lyophilization)	Stabilizes nanoparticles post-preparation for enhanced re-dispersibility.	Maintained structural integrity and bioactivity of peptides and vaccines during storage.	[76,99,108]
Polydopamine-Assisted Surface Modification	Adds adhesion and osteointegration layers to scaffolds or nanoparticles.	Enhanced osteogenic differentiation and antibacterial properties in bone scaffolds.	[59,69,92]
Porogen-Assisted Microsphere Fabrication	Introduces porogens like Ca(OH)2 for controlled release and peptide stability.	Reduced acylation during degradation and enhanced initial release for sustained delivery.	[44,112]
Coating with Biomimetic Membranes	Mimics cellular membranes to enhance immune evasion and targeting capabilities.	Enabled BBB and tumor penetration for targeted glioblastoma and melanoma therapies.	[47,62,94]
Optimization Using Statistical Methods	Response Surface Methodology optimizes encapsulation efficiency, size, and release.	Statistical modeling improved process reproducibility and therapeutic outcomes for advanced nanoparticle formulations.	[74,80,110]
Modified Emulsion Techniques	Improves particle morphology, release profiles, and stability through solvent and surfactant variations.	Enhanced ocular and renal delivery of anti-inflammatory and targeted therapeutic agents.	[1,61,75,80]
Nanocomposites and Hybrid Scaffolds	Combined PLGA with graphene oxide, gold nanoparticles, and peptides for enhanced properties.	Increased conductivity, mechanical strength, and bioactivity for neural and bone tissue engineering.	[60,92,103,104]
Microspheres for Sustained Release	Prolonged drug delivery with optimized solvent evaporation methods and process parameters.	Achieved sustained release profiles for hormone therapies, cancer vaccines, and neuroprotective peptides.	[1,12,98,116,117]
Cell-Penetrating Peptide (CPP) Functionalization	Post-preparation modification for targeted delivery to hard-to-reach tissues.	Improved BBB and intracellular targeting for Alzheimer’s, cancer, and gene therapy applications.	[5,26,29,56]
Incorporation into Hydrogels	Combines PLGA particles with hydrogels for sustained release, especially in bone and wound healing.	Supported enhanced vascularization, osteogenesis, and infection control in complex tissue engineering setups.	[19,36,69,95]
pH-Controlled Microsphere Systems	Buffering agents and pH adjustments prevent acylation and optimize release.	Sustained peptide integrity during acidic degradation while extending drug stability.	[44,93,112]
Drug/Polymer Conjugates	Functionalizes PLGA for localized action through direct conjugation of bioactive molecules.	Improved targeting and bioactivity in bone and vascular regeneration applications.	[91,92,118]
Layer-by-Layer Assembly	Functionalizes nanoparticles for sequential drug release in multi-drug delivery systems.	Achieved synergistic cancer treatment and infection control with controlled peptide/drug layering.	[59,115,119]

**Table 4:** Key physicochemical features in PLGA nanocarriers.

Physicochemical Properties	Key Patterns	Enhanced Observations	Reference
Size (Nanoparticles and Microneedles)	Nanoscale sizes (50–500 nm) dominate for drug delivery; micron-sizes used for vaccines and long-acting depot systems.	Size influences tissue penetration, stability, and drug release. Larger particles (~350 nm) showed minimal acylation over 50 days.	[2,9,24,80,98]
Polydispersity Index (PDI)	Low PDI (<0.2) ensures homogeneity, critical for reproducibility and stability.	Achieved through advanced formulation techniques like Box–Behnken and nanoprecipitation methods.	[9,80,107,110,122]
Surface Charge (Zeta Potential)	Positive zeta potential enhances cellular uptake; negative zeta potential improves colloidal stability.	CPPs and PEGylation fine-tuned surface charges (−46 to +20 mV) for specific targeting and reduced off-target effects.	[26,29,30,55,110]
Encapsulation Efficiency	High efficiency (60–96%) achieved with optimized double-emulsion, nanoprecipitation, or ion-pairing techniques.	Ion-pairing methods significantly reduced burst release, ensuring steady peptide and protein delivery.	[9,11,12,40]
Release Profiles	Sustained release from weeks to months, often tri-phasic (burst, diffusion, erosion).	Achieved with porogen incorporation (e.g., Ca(OH)_2_) or end-capping adjustments; self-immolative strategies minimized acylation.	[14,43,44,69,77]
Stability	PEGylation, freeze-drying, and hybrid matrices improved nanoparticle stability and re-dispersibility.	Stable peptide release achieved over 60 days with hydrophilic gels and PEG-functionalized surfaces.	[19,35,76,108]
Specific Surface Functionalization	Surface modifications (e.g., CPPs, PEGylation, polydopamine coating) improved targeting and tissue compatibility.	Polydopamine coating enhanced osteogenic and antibacterial properties, while CPPs ensured BBB penetration and intracellular uptake.	[25,26,39,69,91]
Biocompatibility and Cytotoxicity	Biocompatibility maintained across applications; formulations showed low or no cytotoxicity.	Tested on various cell lines (e.g., TR146, MG63) and in vivo; no inflammatory responses observed with long-term use.	[16,23,64,123]
Mechanical Properties (Scaffolds and Films)	Enhanced tensile strength and hydrophilicity with materials like graphene oxide, hydroxyapatite, and gelatin blends.	Supported bone regeneration and wound healing with biocompatible, porous scaffolds designed for cellular infiltration and tissue growth.	[38,60,92,103]
Controlled Degradation	Acid-terminated PLGA provides faster degradation; ester-terminated offers longer stability.	Peptides retained activity for up to 60 days; pH buffering minimized peptide degradation during PLGA hydrolysis.	[10,14,77,112]
Targeting Efficiency	Enhanced tissue-specific delivery using erythrocyte membranes, folic acid, or renal-targeting peptides.	BBB crossing achieved with erythrocyte-coated or CPP-functionalized nanoparticles; renal-specific accumulation improved Asiatic acid therapy.	[4,35,61,62]
Adjuvant Effects	Some nanoparticles displayed self-adjuvant properties, reducing need for external adjuvants in vaccines.	Multi-epitope vaccines induced robust IgG, T-cell responses, and cytokine production.	[24,31,32]
Hydrophobic/Hydrophilic Balancing	Ion-pairing and surfactants balanced hydrophilic drug encapsulation and hydrophobic PLGA interaction.	Achieved sustained release for hydrophilic peptides like insulin, exenatide, and octreotide.	[11,40,80,106]
Porosity and Pore Size	Porosity influenced release rates, with microporous scaffolds showing enhanced drug diffusion.	Interconnected pores (30–220 μm) supported osteoblast proliferation and angiogenesis.	[49,77,112]
Self-Assembly and Nanoarchitecture	Self-assembling peptides like RADA16 enhanced scaffold and particle bioactivity.	Improved neural and vascular tissue regeneration through controlled topography and bioactive coatings.	[51,52,114]

**Table 5:** Evaluation metrics and methods for peptide-loaded PLGA nanocarriers.

Testing/Evaluation Focus	Key Observations and Patterns	Reference
Physicochemical Characterization	- Particle size, polydispersity index (PDI), surface charge (zeta potential), and morphology were consistently characterized. - Techniques such as SEM, TEM, DLS, and FTIR were routinely employed.	[9,28,39,65,129]
Encapsulation Efficiency and Drug Loading	- High encapsulation efficiency (>80% in many cases) was a critical success factor. - Optimized formulation methods (e.g., double-emulsion, microfluidics) enhanced consistency and reduced variability.	[11,12,13,44,53,77]
Release Profile Analysis	- Sustained release profiles were a key feature, often spanning weeks to months. - Tri-phasic (burst, lag, erosion) or bi-phasic release was observed, depending on polymer type and drug.	[10,14,15,43,77,93]
In Vitro Drug Release Kinetics	- Advanced models (e.g., Higuchi, Korsmeyer–Peppas) and dissolution apparatus were frequently used. - Predictive IVIVC models helped bridge in vitro data to in vivo outcomes.	[11,13,22,54,77]
Cellular Uptake and Internalization	- Tested using confocal microscopy, flow cytometry, and endocytosis assays. - Surface modifications like enhanced CPP uptake were critical for BBB penetration and intracellular delivery.	[26,28,35,47,57,62]
Toxicity and Cytocompatibility	- Non-toxic profiles were confirmed via MTT, LDH, and ROS assays across cell lines. - Hemolysis and irritation tests were conducted for systemic applications.	[23,59,75,81,120,123]
Immunogenicity and Vaccine Efficacy	- Immune responses were tested using cytokine profiling, IgG/IgA/IFN-γ measurements, and survival studies in animal models. - Self-adjuvant properties were observed in some formulations.	[30,31,32,66,127,130]
Antimicrobial and Antibiofilm Activity	- MIC, CFU count reduction, and biofilm disruption were commonly used metrics. - Synergistic effects with photothermal therapy or peptides like Dermaseptin were noted.	[20,21,55,63,64]
Wound Healing and Tissue Regeneration	- Parameters like granulation tissue, collagen deposition, angiogenesis, and cell migration were tested. - Significant results were noted with osteogenic or angiogenic peptides.	[18,23,37,60,81,91]
Neuronal Differentiation and Growth	- Neural scaffolds demonstrated improved alignment, axonal regeneration, and electrical stimulation response. - IKVAV and RADA16 peptides showed consistent efficacy.	[51,52,85,104,131]
Bone Regeneration and Osteogenic Activity	- Enhanced ALP, collagen I, and osteocalcin expression validated osteogenic properties. - Mechanical properties like compressive strength were key for scaffold applications.	[18,19,36,38,92,105]
Cancer Therapy Efficacy	- Tumor targeting was validated via biodistribution, apoptosis assays, and survival studies. - Functionalized nanoparticles improved therapeutic indices.	[2,6,7,47,84,119]
Blood Brain Barrier (BBB) Penetration	- Crossing efficiency was tested via in vitro BBB models and in vivo biodistribution. - CPP-functionalized systems showed marked improvements in brain delivery.	[25,27,28,29,62]
Allergen Response and Immune Tolerance	- Models for cow’s milk allergy demonstrated reduced IgE and cytokine responses. - Immune tolerance was achieved with peptide fragments in PLGA systems.	[70,72,79,130]
Biophysical Stability and Performance	- Freeze-drying, pH sensitivity, and shelf stability were tested for long-term usability. - Nanoparticles retained functionality post-nebulization and thermal stress.	[40,76,93,108,113]
Thermal and Mechanical Testing	- Scaffolds and films were assessed for hydrophilicity, mechanical strength, and degradation under physiological conditions.	[49,60,86,91,92]
Gene and Protein Delivery Efficiency	- Transfection efficiencies were high with PEG/PLGA and CPP systems. - Gene delivery efficacy correlated with surface modification and encapsulation strategies.	[35,45,56,74,89]
Hypoglycemic and Diabetes-Related Tests	- Bioavailability of insulin formulations was enhanced with CPPs. - Hypoglycemic effect was significant in in vivo models.	[16,29,102]

**Table 6:** Proven benefits of peptide-loaded PLGA products.

Benefit	Description	Supporting Factors	Reference
Sustained Drug Release	Prolonged therapeutic effect with tri-phasic or bi-phasic release profiles.	Achieved using porogens, end-capping, or hybrid polymer matrices.	[14,22,43,69,77]
Enhanced Stability	Improved peptide stability against degradation and acylation.	PEGylation, ion-pairing, and freeze-drying methods.	[11,19,35,76]
Reduced Dosing Frequency	Long-acting formulations minimize the need for frequent administrations.	Microspheres and depot systems for chronic disease management.	[13,42,77,78]
Improved Targeting and Biodistribution	Tissue-specific delivery reduces systemic toxicity and improves therapeutic outcomes.	Functionalization with CPPs, T-cell receptors, and ligands.	[25,29,61,62]
Biocompatibility and Low Toxicity	Safe for use in various systems with minimal inflammatory or cytotoxic responses.	Validated through in vitro and in vivo toxicity assays.	[16,23,64,123]
Facilitation of BBB Penetration	Effective delivery of therapeutic agents to the brain.	CPP-functionalized nanoparticles and erythrocyte-mimetic coatings.	[25,27,29,62]
Multifunctional Capabilities	Combines therapeutic, diagnostic, and regenerative functions in a single platform.	Integration of antimicrobial, osteogenic, and angiogenic properties.	[19,23,91,114]
Efficient Immune Response Activation	Strong antigen-specific responses for vaccines and immunotherapies.	Sustained antigen release and self-adjuvant nanoparticle designs.	[31,32,33,66]
Antimicrobial Efficacy	Effective against multidrug-resistant bacteria and biofilm-related infections.	Use of antimicrobial peptides and synergistic photothermal therapies.	[20,55,63,64]
Versatility Across Delivery Routes	Applicable to oral, buccal, intranasal, ocular, and parenteral delivery systems.	Mucoadhesive formulations and surface functionalization techniques.	[64,76,102,110]
Customizable Release Profiles	Controlled drug release tailored for specific therapeutic needs.	Techniques like hydrophobic ion-pairing and hybrid polymer blends.	[10,11,14,77]
Regenerative Medicine Applications	Promotes angiogenesis, osteogenesis, and neural regeneration for tissue repair.	Peptide-functionalized scaffolds and self-assembling systems.	[18,19,52,59]
Reduced Burst Release	Minimizes initial drug loss, improving therapeutic consistency.	Achieved using stabilizers, ion-pairing, and emulsion techniques.	[11,43,44]
Scalability Potential	Advanced techniques like microfluidics and electrospraying improve reproducibility and scalability.	Essential for industrial applications and large-scale production.	[40,41,42]
Proven Integration with Diagnostics	Enables real-time monitoring through multifunctional nanocarriers.	Use of imaging agents and dual-purpose formulations.	[91,99,125]

**Table 7:** Comparative analysis of applications, advantages, and challenges of PLGA-based systems.

Application	Advantages	Challenges	References
Antibiofilm and Antimicrobial Peptides	Enhanced infection treatment with prolonged peptide activity.	Limited efficacy in polymicrobial biofilms and potential resistance development.	[9,21,55,64]
Cancer Therapies	Improved targeting and reduced systemic toxicity.	High production costs and complex regulatory approval pathways for functionalized nanoparticles.	[2,6,7,34,39]
Neural Tissue Repair	Promoted neurite outgrowth under electrical stimulation.	Challenges in translating in vitro neural differentiation to clinical applications.	[51,85,104,114]
Vaccine Development	Enhanced immunogenicity and sustained antigen release.	Risks of unexpected immune reactions or reduced stability during storage.	[24,30,31,32,66]
Autoimmune Disease Treatment	Reduced severity of conditions like encephalomyelitis.	Long-term effects and precise dose optimization remain underexplored.	[15,79,117]
Wound Healing	Accelerated healing via angiogenesis and antimicrobial activity.	Dependence on local microenvironment and variation in individual responses.	[23,55,59,81,100]
Obesity Therapy (Gene Delivery)	Enhanced cellular uptake and serum stability for antisense oligonucleotides.	Requires optimization to avoid off-target gene modulation.	[73]
Ischemic Injury Treatment	Prolonged angiogenesis and blood flow restoration.	Risks of localized inflammation or overstimulation of angiogenic pathways.	[83,89]
Alzheimer’s Therapy	Brain penetration with reduced beta-amyloid deposits.	Limited long-term efficacy studies and scalability issues.	[3,4,8,27]
Pulmonary Gene Delivery	Efficient cellular uptake and eGFP expression in lung cells.	Risks of immune responses to plasmid DNA or CPP-modified nanoparticles.	[28,56,57]
Cardiovascular Therapy	Specific endothelial targeting and improved eNOS phosphorylation.	Limited long-term clinical data on cardiovascular outcomes.	[84,106]
Ocular Therapies	Improved ocular penetration with no cytotoxicity.	Maintaining prolonged therapeutic levels in dynamic ocular environments.	[17,75,76,108]
Bone Tissue Engineering	Controlled release of osteogenic factors; multifunctional scaffold integration.	Manufacturing complexity for 3D-printed scaffolds with consistent bioactivity.	[18,19,38,60]
Cancer Immunotherapy	Strong CTL responses and enhanced antigen delivery.	Tumor heterogeneity may reduce targeting efficacy.	[33,45,46,127]
Allergy Prevention	Modulation of immune responses and reduction of Th2-driven symptoms.	Risk of unintended immune modulation with prolonged use.	[70,72]
Neurotherapeutics	Efficient BBB penetration and sustained release.	Validation of delivery efficiency across diverse neurological models.	[29,102,109]
Peptide Delivery Systems	Improved stability and reduced acylation risks.	Initial burst release in long-acting injectable systems.	[44,78,94,98]

## Data Availability

No new data were created or analyzed in this study. Data sharing is not applicable to this article.

## References

[1] Andhariya J.V., Jog R., Shen J., Choi S., Wang Y., Zou Y., Burgess D.J. (2019). Development of Level A in vitro-in vivo correlations for peptide loaded PLGA microspheres. J. Control. Release.

[2] Bhowmik A., Chakravarti S., Ghosh A., Shaw R., Bhandary S., Bhattacharyya S., Sen P.C., Ghosh M.K. (2017). Anti-SSTR2 peptide based targeted delivery of potent PLGA encapsulated 3,3′-diindolylmethane nanoparticles through blood brain barrier prevents glioma progression. Oncotarget.

[3] Fan S., Zheng Y., Liu X., Fang W., Chen X., Liao W., Jing X., Lei M., Tao E., Ma Q. (2018). Curcumin-loaded PLGA-PEG nanoparticles conjugated with B6 peptide for potential use in Alzheimer’s disease. Drug Deliv..

[4] Huang N., Lu S., Liu X.G., Zhu J., Wang Y.J., Liu R.T. (2017). PLGA nanoparticles modified with a BBB-penetrating peptide co-delivering Abeta generation inhibitor and curcumin attenuate memory deficits and neuropathology in Alzheimer’s disease mice. Oncotarget.

[5] Mathew A., Fukuda T., Nagaoka Y., Hasumura T., Morimoto H., Yoshida Y., Maekawa T., Venugopal K., Kumar D.S. (2012). Curcumin loaded-PLGA nanoparticles conjugated with Tet-1 peptide for potential use in Alzheimer’s disease. PLoS ONE.

[6] Panda P.K., Jain S.K. (2023). Doxorubicin bearing peptide anchored PEGylated PLGA nanoparticles for the effective delivery to prostate cancer cells. J. Drug Deliv. Sci. Technol..

[7] Priwitaningrum D.L., Jentsch J., Bansal R., Rahimian S., Storm G., Hennink W.E., Prakash J. (2020). Apoptosis-inducing peptide loaded in PLGA nanoparticles induces anti-tumor effects in vivo. Int. J. Pharm..

[8] Saleh S.R., Abd-Elmegied A., Aly Madhy S., Khattab S.N., Sheta E., Elnozahy F.Y., Mehanna R.A., Ghareeb D.A., Abd-Elmonem N.M. (2024). Brain-targeted Tet-1 peptide-PLGA nanoparticles for berberine delivery against STZ-induced Alzheimer’s disease in a rat model: Alleviation of hippocampal synaptic dysfunction, Tau pathology, and amyloidogenesis. Int. J. Pharm..

[9] Ali M., van Gent M.E., de Waal A.M., van Doodewaerd B.R., Bos E., Koning R.I., Cordfunke R.A., Drijfhout J.W., Nibbering P.H. (2023). Physical and Functional Characterization of PLGA Nanoparticles Containing the Antimicrobial Peptide SAAP-148. Int. J. Mol. Sci..

[10] Encinas-Basurto D., Konhilas J.P., Polt R., Hay M., Mansour H.M. (2022). Glycosylated Ang-(1-7) MasR Agonist Peptide Poly Lactic-co-Glycolic Acid (PLGA) Nanoparticles and Microparticles in Cognitive Impairment: Design, Particle Preparation, Physicochemical Characterization, and In Vitro Release. Pharmaceutics.

[11] Liu J., Xu Y., Liu Z., Ren H., Meng Z., Liu K., Liu Z., Yong J., Wang Y., Li X. (2019). A modified hydrophobic ion-pairing complex strategy for long-term peptide delivery with high drug encapsulation and reduced burst release from PLGA microspheres. Eur. J. Pharm. Biopharm..

[12] Arora G., Shukla J., Ghosh S., Maulik S.K., Malhotra A., Bandopadhyaya G. (2012). PLGA nanoparticles for peptide receptor radionuclide therapy of neuroendocrine tumors: A novel approach towards reduction of renal radiation dose. PLoS ONE.

[13] Goel M., Leung D., Famili A., Chang D., Nayak P., Al-Sayah M. (2021). Accelerated in vitro release testing method for a long-acting peptide-PLGA formulation. Eur. J. Pharm. Biopharm..

[14] Tomic I., Vidis-Millward A., Mueller-Zsigmondy M., Cardot J.M. (2016). Setting accelerated dissolution test for PLGA microspheres containing peptide, investigation of critical parameters affecting drug release rate and mechanism. Int. J. Pharm..

[15] Cappellano G., Woldetsadik A.D., Orilieri E., Shivakumar Y., Rizzi M., Carniato F., Gigliotti C.L., Boggio E., Clemente N., Comi C. (2014). Subcutaneous inverse vaccination with PLGA particles loaded with a MOG peptide and IL-10 decreases the severity of experimental autoimmune encephalomyelitis. Vaccine.

[16] Castro P.M., Baptista P., Madureira A.R., Sarmento B., Pintado M.E. (2018). Combination of PLGA nanoparticles with mucoadhesive guar-gum films for buccal delivery of antihypertensive peptide. Int. J. Pharm..

[17] Jiao X., Dong X., Shan H., Qin Z. (2023). Assessing the Efficacy of PLGA-Loaded Antimicrobial Peptide OH-CATH30 Microspheres for the Treatment of Bacterial Keratitis: A Promising Approach. Biomolecules.

[18] Lin Z.Y., Duan Z.X., Guo X.D., Li J.F., Lu H.W., Zheng Q.X., Quan D.P., Yang S.H. (2010). Bone induction by biomimetic PLGA-(PEG-ASP)n copolymer loaded with a novel synthetic BMP-2-related peptide in vitro and in vivo. J. Control. Release.

[19] Wu G., Cao Z.Z., Luo X.L., Wang X.X., Wang S.H., Wang D.L. (2017). Fabrication and Characterization of a PDLSCs/BMP-2-PLGA-NP/RADA Peptide Hydrogel Composite for Bone Repair. J. Biomater. Tissue Eng..

[20] Guo M., Ruan M., Wu J., Ye J., Wang C., Guo Z., Chen W., Wang L., Wu K., Du S. (2025). Poly-tannic acid coated PLGA nanoparticle decorated with antimicrobial peptide for synergistic bacteria treatment and infectious wound healing promotion. Colloids Surf. B Biointerfaces.

[21] Gomez-Sequeda N., Ruiz J., Ortiz C., Urquiza M., Torres R. (2020). Potent and Specific Antibacterial Activity against Escherichia coli O157:H7 and Methicillin Resistant Staphylococcus aureus (MRSA) of G17 and G19 Peptides Encapsulated into Poly-Lactic-Co-Glycolic Acid (PLGA) Nanoparticles. Antibiotics.

[22] Tomic I., Mueller-Zsigmondy M., Vidis-Millward A., Cardot J.M. (2018). In vivo release of peptide-loaded PLGA microspheres assessed through deconvolution coupled with mechanistic approach. Eur. J. Pharm. Biopharm..

[23] Chereddy K.K., Her C.H., Comune M., Moia C., Lopes A., Porporato P.E., Vanacker J., Lam M.C., Steinstraesser L., Sonveaux P. (2014). PLGA nanoparticles loaded with host defense peptide LL37 promote wound healing. J. Control. Release.

[24] Buyukbayraktar H.K., Pelit Arayici P., Ihlamur M., Gokkaya D., Karahan M., Abamor E.S., Topuzogullari M. (2023). Effect of polycation coating on the long-term pulsatile release of antigenic ESAT-6(1-20) peptide from PLGA nanoparticles. Colloids Surf. B Biointerfaces.

[25] Bi Y., Liu L., Lu Y., Sun T., Shen C., Chen X., Chen Q., An S., He X., Ruan C. (2016). T7 Peptide-Functionalized PEG-PLGA Micelles Loaded with Carmustine for Targeting Therapy of Glioma. ACS Appl. Mater. Interfaces.

[26] Feiner-Gracia N., Dols-Perez A., Royo M., Solans C., Garcia-Celma M.J., Fornaguera C. (2018). Cell penetrating peptide grafting of PLGA nanoparticles to enhance cell uptake. Eur. Polym. J..

[27] Khairnar B.D., Padhye A., Madiwal V., Jha A., Jadhav S.H., Rajwade J.M. (2023). Cyclic ß-hairpin peptide loaded PLGA nanoparticles: A potential anti-amyloid therapeutic. Mater. Today Commun..

[28] Vijayan A.N., Indrakumar J., Gomathinayagam S., Gothandam K.M., Korrapati P.S. (2022). Bi-Functional Aspects of Peptide Decorated PLGA Nanocarriers for Enhanced Translocation Across the Blood-Brain Barrier through Macropinocytosis. Macromol. Res..

[29] Yan L., Wang H.Y., Jiang Y.F., Liu J.H., Wang Z., Yang Y.X., Huang S.W., Huang Y.Z. (2013). Cell-Penetrating Peptide-Modified PLGA Nanoparticles for Enhanced Nose-to-Brain Macromolecular Delivery. Macromol. Res..

[30] Du X., Xue J., Jiang M., Lin S., Huang Y., Deng K., Shu L., Xu H., Li Z., Yao J. (2021). A Multiepitope Peptide, rOmp22, Encapsulated in Chitosan-PLGA Nanoparticles as a Candidate Vaccine Against Acinetobacter baumannii Infection. Int. J. Nanomed..

[31] Heng W.T., Lim H.X., Tan K.O., Poh C.L. (2023). Validation of Multi-epitope Peptides Encapsulated in PLGA Nanoparticles Against Influenza A Virus. Pharm. Res..

[32] Roozbehani M., Falak R., Mohammadi M., Hemphill A., Razmjou E., Meamar A.R., Masoori L., Khoshmirsafa M., Moradi M., Gharavi M.J. (2018). Characterization of a multi-epitope peptide with selective MHC-binding capabilities encapsulated in PLGA nanoparticles as a novel vaccine candidate against Toxoplasma gondii infection. Vaccine.

[33] Varypataki E.M., Silva A.L., Barnier-Quer C., Collin N., Ossendorp F., Jiskoot W. (2016). Synthetic long peptide-based vaccine formulations for induction of cell mediated immunity: A comparative study of cationic liposomes and PLGA nanoparticles. J. Control. Release.

[34] Nie X., Liu Y., Li M., Yu X., Yuan W., Huang S., Ren D., Wang Y., Wang Y. (2020). SP94 Peptide-Functionalized PEG-PLGA Nanoparticle Loading with Cryptotanshinone for Targeting Therapy of Hepatocellular Carcinoma. AAPS PharmSciTech.

[35] Paulino da Silva Filho O., Ali M., Nabbefeld R., Primavessy D., Bovee-Geurts P.H., Grimm S., Kirchner A., Wiesmuller K.H., Schneider M., Walboomers X.F. (2021). A comparison of acyl-moieties for noncovalent functionalization of PLGA and PEG-PLGA nanoparticles with a cell-penetrating peptide. RSC Adv..

[36] Cai Q., Qiao C., Ning J., Ding X., Wang H., Zhou Y. (2019). A Polysaccharide-based Hydrogel and PLGA Microspheres for Sustained P24 Peptide Delivery: An In vitro and In vivo Study Based on Osteogenic Capability. Chem. Res. Chin. Univ..

[37] Shafiq M., Yuan Z., Rafique M., Aishima S., Jing H., Yuqing L., Ijima H., Jiang S., Mo X. (2023). Combined effect of SDF-1 peptide and angiogenic cues in co-axial PLGA/gelatin fibers for cutaneous wound healing in diabetic rats. Colloids Surf. B Biointerfaces.

[38] Wang X., Chen W., Chen Z., Li Y., Wu K., Song Y. (2022). Preparation of 3D Printing PLGA Scaffold with BMP-9 and P-15 Peptide Hydrogel and Its Application in the Treatment of Bone Defects in Rabbits. Contrast Media Mol. Imaging.

[39] Chen J., Li S., Shen Q. (2012). Folic acid and cell-penetrating peptide conjugated PLGA-PEG bifunctional nanoparticles for vincristine sulfate delivery. Eur. J. Pharm. Sci..

[40] Schlosser C.S., Morris C.J., Brocchini S., Williams G.R. (2024). Hydrophobic ion pairing as a novel approach to co-axial electrospraying of peptide-PLGA particles. Int. J. Pharm..

[41] Streck S., Neumann H., Nielsen H.M., Rades T., McDowell A. (2019). Comparison of bulk and microfluidics methods for the formulation of poly-lactic-co-glycolic acid (PLGA) nanoparticles modified with cell-penetrating peptides of different architectures. Int. J. Pharm. X.

[42] Zhang C., Yang L., Wan F., Bera H., Cun D., Rantanen J., Yang M. (2020). Quality by design thinking in the development of long-acting injectable PLGA/PLA-based microspheres for peptide and protein drug delivery. Int. J. Pharm..

[43] Balmert S.C., Zmolek A.C., Glowacki A.J., Knab T.D., Rothstein S.N., Wokpetah J.M., Fedorchak M.V., Little S.R. (2015). Positive Charge of “Sticky” Peptides and Proteins Impedes Release From Negatively Charged PLGA Matrices. J. Mater. Chem. B.

[44] Xiao P., Qi P., Chen J., Song Z., Wang Y., He H., Tang X., Wang P. (2020). The effect of polymer blends on initial release regulation and in vitro-in vivo relationship of peptides loaded PLGA-Hydrogel Microspheres. Int. J. Pharm..

[45] Silva A.L., Rosalia R.A., Sazak A., Carstens M.G., Ossendorp F., Oostendorp J., Jiskoot W. (2013). Optimization of encapsulation of a synthetic long peptide in PLGA nanoparticles: Low-burst release is crucial for efficient CD8(+) T cell activation. Eur. J. Pharm. Biopharm..

[46] Zhang N., Li J., Gao W., Zhu W., Yan J., He Z., Li L., Wu F., Pu Y., He B. (2022). Co-Delivery of Doxorubicin and Anti-PD-L1 Peptide in Lipid/PLGA Nanocomplexes for the Chemo-Immunotherapy of Cancer. Mol. Pharm..

[47] Yaman S., Ramachandramoorthy H., Oter G., Zhukova D., Nguyen T., Sabnani M.K., Weidanz J.A., Nguyen K.T. (2020). Melanoma Peptide MHC Specific TCR Expressing T-Cell Membrane Camouflaged PLGA Nanoparticles for Treatment of Melanoma Skin Cancer. Front. Bioeng. Biotechnol..

[48] Sathya S., Shanmuganathan B., Saranya S., Vaidevi S., Ruckmani K., Pandima Devi K. (2018). Phytol-loaded PLGA nanoparticle as a modulator of Alzheimer’s toxic Abeta peptide aggregation and fibrillation associated with impaired neuronal cell function. Artif. Cells Nanomed. Biotechnol..

[49] Wang L., Li C.Y., He P., Fu L., Zhou Y.M., Chen X.S. (2011). Preparation and Bioactivities of PLGA/Nano-hydroxyapatite Scaffold Containing Chitosan Microspheres for Controlled Delivery of Mutifuncational Peptide-adrenomedullin. Chem. J. Chin. Univ..

[50] Zhang S.B., Zhang Z., Yu M.N., Liu T.B., Wang J.J., Cai Q., Chen L., He C.L., Meng W.Y., Chen X.S. (2014). Biodegradable PLGA Microspheres for Controlled Delivery of Parathyroid Hormone Related Peptide. Acta Polym. Sin..

[51] Nune M., Krishnan U.M., Sethuraman S. (2015). Decoration of PLGA electrospun nanofibers with designer self-assembling peptides: A “Nano-on-Nano” concept. RSC Adv..

[52] Nune M., Krishnan U.M., Sethuraman S. (2016). PLGA nanofibers blended with designer self-assembling peptides for peripheral neural regeneration. Mater. Sci. Eng. C Mater. Biol. Appl..

[53] Derman S., Mustafaeva Z.A., Abamor E.S., Bagirova M., Allahverdiyev A. (2015). Preparation, characterization and immunological evaluation: Canine parvovirus synthetic peptide loaded PLGA nanoparticles. J. Biomed. Sci..

[54] Ucar B., Acar T., Arayici P.P., Derman S. (2021). A nanotechnological approach in the current therapy of COVID-19: Model drug oseltamivir-phosphate loaded PLGA nanoparticles targeted with spike protein binder peptide of SARS-CoV-2. Nanotechnology.

[55] Ramoa A.M., Campos F., Moreira L., Teixeira C., Leiro V., Gomes P., das Neves J., Martins M.C.L., Monteiro C. (2023). Antimicrobial peptide-grafted PLGA-PEG nanoparticles to fight bacterial wound infections. Biomater. Sci..

[56] Gomes Dos Reis L., Lee W.H., Svolos M., Moir L.M., Jaber R., Windhab N., Young P.M., Traini D. (2019). Nanotoxicologic Effects of PLGA Nanoparticles Formulated with a Cell-Penetrating Peptide: Searching for a Safe pDNA Delivery System for the Lungs. Pharmaceutics.

[57] Gomes Dos Reis L., Lee W.H., Svolos M., Moir L.M., Jaber R., Engel A., Windhab N., Young P.M., Traini D. (2020). Delivery of pDNA to lung epithelial cells using PLGA nanoparticles formulated with a cell-penetrating peptide: Understanding the intracellular fate. Drug Dev. Ind. Pharm..

[58] Malik S., Slack F.J., Bahal R. (2020). Formulation of PLGA nanoparticles containing short cationic peptide nucleic acids. Methodsx.

[59] Zhang Z.Y., Zhou S.C., Zhang Y.Z., Wu D.K., Yang X.Y. (2020). The dual delivery of growth factors and antimicrobial peptide by PLGA/GO composite biofilms to promote skin-wound healing. New J. Chem..

[60] Liu Z.H., Tian G.J., Liu L.N., Li Y.M., Xu S.D., Du Y.Q., Li M.T., Jing W., Wei P.F., Zhao B. (2024). A 3D-printed PLGA/HA composite scaffold modified with fusion peptides to enhance its antibacterial, osteogenic and angiogenic properties in bone defect repair. J. Mater. Res. Technol..

[61] He J., Chen H., Zhou W., Chen M., Yao Y., Zhang Z., Tan N. (2020). Kidney targeted delivery of asiatic acid using a FITC labeled renal tubular-targeting peptide modified PLGA-PEG system. Int. J. Pharm..

[62] Cui Y., Sun J., Hao W., Chen M., Wang Y., Xu F., Gao C. (2020). Dual-Target Peptide-Modified Erythrocyte Membrane-Enveloped PLGA Nanoparticles for the Treatment of Glioma. Front. Oncol..

[63] Cheng Y., Qin J., Huang Y., Wang T. (2022). The antimicrobial effects of PLGA microspheres containing the antimicrobial peptide OP-145 on clinically isolated pathogens in bone infections. Sci. Rep..

[64] Li Y., Na R., Wang X., Liu H., Zhao L., Sun X., Ma G., Cui F. (2017). Fabrication of Antimicrobial Peptide-Loaded PLGA/Chitosan Composite Microspheres for Long-Acting Bacterial Resistance. Molecules.

[65] Çalman F., Pelit Arayıcı P., Büyükbayraktar H.K., Karahan M., Mustafaeva Z., Katsarava R. (2018). Development of Vaccine Prototype Against Zika Virus Disease of Peptide-Loaded PLGA Nanoparticles and Evaluation of Cytotoxicity. Int. J. Pept. Res. Ther..

[66] Dolen Y., Gileadi U., Chen J.L., Valente M., Creemers J.H.A., Van Dinther E.A.W., van Riessen N.K., Jager E., Hruby M., Cerundolo V. (2021). PLGA Nanoparticles Co-encapsulating NY-ESO-1 Peptides and IMM60 Induce Robust CD8 and CD4 T Cell and B Cell Responses. Front. Immunol..

[67] Herrmann V.L., Wieland D.E., Legler D.F., Wittmann V., Groettrup M. (2016). The STEAP1(262-270) peptide encapsulated into PLGA microspheres elicits strong cytotoxic T cell immunity in HLA-A*0201 transgenic mice—A new approach to immunotherapy against prostate carcinoma. Prostate.

[68] Clement S., Anwer A.G., Pires L., Campbell J., Wilson B.C., Goldys E.M. (2021). Radiodynamic Therapy Using TAT Peptide-Targeted Verteporfin-Encapsulated PLGA Nanoparticles. Int. J. Mol. Sci..

[69] Chen L., Shao L., Wang F., Huang Y., Gao F. (2019). Enhancement in sustained release of antimicrobial peptide and BMP-2 from degradable three dimensional-printed PLGA scaffold for bone regeneration. RSC Adv..

[70] Liu M., Thijssen S., Hennink W.E., Garssen J., van Nostrum C.F., Willemsen L.E.M. (2022). Oral pretreatment with beta-lactoglobulin derived peptide and CpG co-encapsulated in PLGA nanoparticles prior to sensitizations attenuates cow’s milk allergy development in mice. Front. Immunol..

[71] Hu F.F., Qi J.P., Lu Y., He H.S., Wu W. (2023). PLGA-based implants for sustained delivery of peptides/proteins: Current status, challenge and perspectives. Chin. Chem. Lett..

[72] Liu M., Thijssen S., van Nostrum C.F., Hennink W.E., Garssen J., Willemsen L.E.M. (2022). Inhibition of cow’s milk allergy development in mice by oral delivery of beta-lactoglobulin-derived peptides loaded PLGA nanoparticles is associated with systemic whey-specific immune silencing. Clin. Exp. Allergy.

[73] Choi D.H., Park Y.S. (2019). Arginine-rich Peptide Coated PLGA Nanoparticles Enhance Polymeric Delivery of Antisense HIF1α-oligonucleotide to Fully Differentiated Stiff Adipocytes. Toxicol. Environ. Health Sci..

[74] Liu C., Xie Y., Li X., Yao X., Wang X., Wang M., Li Z., Cao F. (2021). Folic Acid/Peptides Modified PLGA-PEI-PEG Polymeric Vectors as Efficient Gene Delivery Vehicles: Synthesis, Characterization and Their Biological Performance. Mol. Biotechnol..

[75] Gonzalez-Pizarro R., Parrotta G., Vera R., Sanchez-Lopez E., Galindo R., Kjeldsen F., Badia J., Baldoma L., Espina M., Garcia M.L. (2019). Ocular penetration of fluorometholone-loaded PEG-PLGA nanoparticles functionalized with cell-penetrating peptides. Nanomedicine.

[76] Galindo-Camacho R.M., Haro I., Gomara M.J., Espina M., Fonseca J., Martins-Gomes C., Camins A., Silva A.M., Garcia M.L., Souto E.B. (2023). Cell penetrating peptides-functionalized Licochalcone-A-loaded PLGA nanoparticles for ocular inflammatory diseases: Evaluation of in vitro anti-proliferative effects, stabilization by freeze-drying and characterization of an in-situ forming gel. Int. J. Pharm..

[77] Ghassemi A.H., van Steenbergen M.J., Barendregt A., Talsma H., Kok R.J., van Nostrum C.F., Crommelin D.J., Hennink W.E. (2012). Controlled release of octreotide and assessment of peptide acylation from poly(D,L-lactide-co-hydroxymethyl glycolide) compared to PLGA microspheres. Pharm. Res..

[78] Zhang C., Wu L., Tao A., Bera H., Tang X., Cun D., Yang M. (2021). Formulation and in vitro characterization of long-acting PLGA injectable microspheres encapsulating a peptide analog of LHRH. J. Mater. Sci. Technol..

[79] Liu M., Feng D., Liang X., Li M., Yang J., Wang H., Pang L., Zhou Z., Yang Z., Kong D. (2020). Old Dog New Tricks: PLGA Microparticles as an Adjuvant for Insulin Peptide Fragment-Induced Immune Tolerance against Type 1 Diabetes. Mol. Pharm..

[80] Tu S.C., Mai S.T., Shu D., Huang Y.X., Nie Z.H., Wang Y., Yang W.L. (2024). Microfluidic-based preparation of PLGA microspheres facilitating peptide sustained-release. Mater. Lett..

[81] Ramezanpour S., Tavatoni P., Akrami M., Navaei-Nigjeh M., Shiri P. (2022). Potential Wound Healing of PLGA Nanoparticles Containing a Novel L-Carnitine-GHK Peptide Conjugate. J. Nanomater..

[82] Esfandyari-Manesh M., Abdi M., Talasaz A.H., Ebrahimi S.M., Atyabi F., Dinarvand R. (2020). S2P peptide-conjugated PLGA-Maleimide-PEG nanoparticles containing Imatinib for targeting drug delivery to atherosclerotic plaques. DARU J. Pharm. Sci..

[83] Choi Y.H., Heo S.C., Kwon Y.W., Kim H.D., Kim S.H., Jang I.H., Kim J.H., Hwang N.S. (2015). Injectable PLGA microspheres encapsulating WKYMVM peptide for neovascularization. Acta Biomater..

[84] Imanparast F., Faramarzi M.A., Vatannejad A., Paknejad M., Deiham B., Kobarfard F., Amani A., Doosti M. (2017). mZD7349 peptide-conjugated PLGA nanoparticles directed against VCAM-1 for targeted delivery of simvastatin to restore dysfunctional HUVECs. Microvasc. Res..

[85] Ozcicek I., Aysit N., Balcikanli Z., Ayturk N.U., Aydeger A., Baydas G., Aydin M.S., Altintas E., Erim U.C. (2024). Development of BDNF/NGF/IKVAV Peptide Modified and Gold Nanoparticle Conductive PCL/PLGA Nerve Guidance Conduit for Regeneration of the Rat Spinal Cord Injury. Macromol. Biosci..

[86] Shin Y.C., Kim J., Kim S.E., Song S.J., Hong S.W., Oh J.W., Lee J., Park J.C., Hyon S.H., Han D.W. (2017). RGD peptide and graphene oxide co-functionalized PLGA nanofiber scaffolds for vascular tissue engineering. Regen. Biomater..

[87] Jiang T., Singh B., Li H.S., Kim Y.K., Kang S.K., Nah J.W., Choi Y.J., Cho C.S. (2014). Targeted oral delivery of BmpB vaccine using porous PLGA microparticles coated with M cell homing peptide-coupled chitosan. Biomaterials.

[88] Wang P.Y., Wu T.H., Tsai W.B., Kuo W.H., Wang M.J. (2013). Grooved PLGA films incorporated with RGD/YIGSR peptides for potential application on skeletal muscle tissue engineering. Colloids Surf. B Biointerfaces.

[89] Park J.S., Yang H.N., Yi S.W., Kim J.H., Park K.H. (2016). Neoangiogenesis of human mesenchymal stem cells transfected with peptide-loaded and gene-coated PLGA nanoparticles. Biomaterials.

[90] Egusquiaguirre S.P., Manguan-Garcia C., Perona R., Pedraz J.L., Hernandez R.M., Igartua M. (2014). Development and validation of a rapid HPLC method for the quantification of GSE4 peptide in biodegradable PEI-PLGA nanoparticles. J. Chromatogr. B Analyt. Technol. Biomed. Life Sci..

[91] Pan H., Zheng Q., Yang S., Guo X. (2014). Effects of functionalization of PLGA-[Asp-PEG]n copolymer surfaces with Arg-Gly-Asp peptides, hydroxyapatite nanoparticles, and BMP-2-derived peptides on cell behavior in vitro. J. Biomed. Mater. Res. A.

[92] Wang Z., Chen L., Wang Y., Chen X., Zhang P. (2016). Improved Cell Adhesion and Osteogenesis of op-HA/PLGA Composite by Poly(dopamine)-Assisted Immobilization of Collagen Mimetic Peptide and Osteogenic Growth Peptide. ACS Appl. Mater. Interfaces.

[93] Liu J., Xu Y., Wang Y., Ren H., Meng Z., Liu K., Liu Z., Huang H., Li X. (2019). Proton Oriented-”Smart Depot” for Responsive Release of Ca(2+) to Inhibit Peptide Acylation in PLGA Microspheres. Pharm. Res..

[94] Zhang Y., Schwendeman S.P. (2012). Minimizing acylation of peptides in PLGA microspheres. J. Control. Release.

[95] Wang M., Guo X., Tan R., She Z., Feng Q. (2013). Effect of stabilizers on bioactivity of peptide-24 in PLGA microspheres. Med. Chem..

[96] Senturk F., Cakmak S. (2023). Fabrication of curcumin-loaded magnetic PEGylated-PLGA nanocarriers tagged with GRGDS peptide for improving anticancer activity. Methodsx.

[97] Wang J.H., Li Y., Jing J., Yue H.L., Zhang L.L., Hua W., Li N., Liu X., Han J.A. (2021). Practical evaluations of bioactive peptide-modified Fluorapatite/PLGA multifunctional nano-clustery composite against for root caries restorations to inhibit periodontitis-related pathogens in periodontitis care. Mater. Res. Express.

[98] Shirangi M., Najafi M., Rijkers D.T., Kok R.J., Hennink W.E., van Nostrum C.F. (2016). Inhibition of Octreotide Acylation Inside PLGA Microspheres by Derivatization of the Amines of the Peptide with a Self-Immolative Protecting Group. Bioconjug. Chem..

[99] Liu M., Lau C.Y.J., Cabello I.T., Garssen J., Willemsen L.E.M., Hennink W.E., van Nostrum C.F. (2023). Live Cell Imaging by Forster Resonance Energy Transfer Fluorescence to Study Trafficking of PLGA Nanoparticles and the Release of a Loaded Peptide in Dendritic Cells. Pharmaceuticals.

[100] Xu S., Tian G., Zhi M., Liu Z., Du Y., Lu X., Li M., Bai J., Li X., Deng J. (2024). Functionalized PLGA Microsphere Loaded with Fusion Peptide for Therapy of Bone Defects. ACS Biomater. Sci. Eng..

[101] Cai H., Liang Z., Huang W., Wen L., Chen G. (2017). Engineering PLGA nano-based systems through understanding the influence of nanoparticle properties and cell-penetrating peptides for cochlear drug delivery. Int. J. Pharm..

[102] Zhu S., Chen S., Gao Y., Guo F., Li F., Xie B., Zhou J., Zhong H. (2016). Enhanced oral bioavailability of insulin using PLGA nanoparticles co-modified with cell-penetrating peptides and Engrailed secretion peptide (Sec). Drug Deliv..

[103] Shin Y.C., Lee J.H., Kim M.J., Hong S.W., Kim B., Hyun J.K., Choi Y.S., Park J.C., Han D.W. (2015). Stimulating effect of graphene oxide on myogenesis of C2C12 myoblasts on RGD peptide-decorated PLGA nanofiber matrices. J. Biol. Eng..

[104] Aydeger A., Aysit N., Baydas G., Cakici C., Erim U.C., Arpa M.D., Ozcicek I. (2023). Design of IKVAV peptide/gold nanoparticle decorated, micro/nano-channeled PCL/PLGA film scaffolds for neuronal differentiation and neurite outgrowth. Biomater. Adv..

[105] Pan H., Hao S., Zheng Q., Li J., Zheng J., Hu Z., Yang S., Guo X., Yang Q. (2013). Bone induction by biomimetic PLGA copolymer loaded with a novel synthetic RADA16-P24 peptide in vivo. Mater. Sci. Eng. C Mater. Biol. Appl..

[106] Ma C., Wei T., Hua Y., Wang Z., Zhang L. (2021). Effective Antitumor of Orally Intestinal Targeting Penetrating Peptide-Loaded Tyroserleutide/PLGA Nanoparticles in Hepatocellular Carcinoma. Int. J. Nanomed..

[107] Li J., Feng L., Fan L., Zha Y., Guo L., Zhang Q., Chen J., Pang Z., Wang Y., Jiang X. (2011). Targeting the brain with PEG-PLGA nanoparticles modified with phage-displayed peptides. Biomaterials.

[108] Galindo R., Sanchez-Lopez E., Gomara M.J., Espina M., Ettcheto M., Cano A., Haro I., Camins A., Garcia M.L. (2022). Development of Peptide Targeted PLGA-PEGylated Nanoparticles Loading Licochalcone-A for Ocular Inflammation. Pharmaceutics.

[109] Zhao X., Ni S., Song Y., Hu K. (2023). Intranasal delivery of Borneol/R8dGR peptide modified PLGA nanoparticles co-loaded with curcumin and cisplatin alleviate hypoxia in pediatric brainstem glioma which improves the synergistic therapy. J. Control. Release.

[110] Bisht R., Rupenthal I.D. (2018). PLGA nanoparticles for intravitreal peptide delivery: Statistical optimization, characterization and toxicity evaluation. Pharm. Dev. Technol..

[111] Song X., Li X., Wang F., Wang L., Lv L., Xie Q., Zhang X., Shao X. (2022). Bioinspired Protein/Peptide Loaded 3D Printed PLGA Scaffold Promotes Bone Regeneration. Front. Bioeng. Biotechnol..

[112] Liu J., Xu Y., Wang Y., Ren H., Meng Z., Liu K., Liu Z., Huang H., Li X. (2019). Effect of inner pH on peptide acylation within PLGA microspheres. Eur. J. Pharm. Sci..

[113] Chintapula U., Yang S., Nguyen T., Li Y., Jaworski J., Dong H., Nguyen K.T. (2022). Supramolecular Peptide Nanofiber/PLGA Nanocomposites for Enhancing Pulmonary Drug Delivery. ACS Appl. Mater. Interfaces.

[114] Shin Y.C., Lee J.H., Jin O.S., Lee E.J., Jin L.H., Kim C.S., Hong S.W., Han D.W., Kim C., Oh J.W. (2015). RGD peptide-displaying M13 bacteriophage/PLGA nanofibers as cell-adhesive matrices for smooth muscle cells. J. Korean Phys. Soc..

[115] Wu C., Wang C., Sun L., Xu K., Zhong W. (2020). PLGA nanoparticle-reinforced supramolecular peptide hydrogels for local delivery of multiple drugs with enhanced synergism. Soft Matter.

[116] Roberts R., Smyth J.W., Will J., Roberts P., Grek C.L., Ghatnekar G.S., Sheng Z., Gourdie R.G., Lamouille S., Foster E.J. (2020). Development of PLGA nanoparticles for sustained release of a connexin43 mimetic peptide to target glioblastoma cells. Mater. Sci. Eng. C Mater. Biol. Appl..

[117] Jin L., Pan Y., Pham A.C., Boyd B.J., Norton R.S., Nicolazzo J.A. (2021). Prolonged Plasma Exposure of the Kv1.3-Inhibitory Peptide HsTX1[R14A] by Subcutaneous Administration of a Poly(Lactic-co-Glycolic Acid) (PLGA) Microsphere Formulation. J. Pharm. Sci..

[118] Sneh-Edri H., Likhtenshtein D., Stepensky D. (2011). Intracellular targeting of PLGA nanoparticles encapsulating antigenic peptide to the endoplasmic reticulum of dendritic cells and its effect on antigen cross-presentation in vitro. Mol. Pharm..

[119] Mohan A.K., Minsa M., Kumar T.R.S., Kumar G.S.V. (2022). Multi-Layered PLGA-PEI Nanoparticles Functionalized with TKD Peptide for Targeted Delivery of Pep5 to Breast Tumor Cells and Spheroids. Int. J. Nanomed..

[120] Koyuncu R., Duruksu G., Ozcelik B., Mert S., Yazir Y. (2023). Effect of Peptide-Lipid Conjugates Loaded PLGA Nanoparticles Against Cancer Cells In-Vitro. J. Biomed. Nanotechnol..

[121] Margaroni M., Agallou M., Kontonikola K., Karidi K., Kammona O., Kiparissides C., Gaitanaki C., Karagouni E. (2016). PLGA nanoparticles modified with a TNFalpha mimicking peptide, soluble Leishmania antigens and MPLA induce T cell priming in vitro via dendritic cell functional differentiation. Eur. J. Pharm. Biopharm..

[122] Derman S., Akdeste Z.M., Ates S.C., Mansuroglu B., Kizilbey K., Bagirova M., Allahverdiyev A. (2017). The study of syntetic peptide loaded plga nanoparticles cytotoxicity in vitro. Fresen. Environ. Bull..

[123] Santos A.P.d., Oliveira R.C.R.d., Louchard B.O., Uchoa A.F.J., Ricardo N.M.P.S., Leal L.K.A.M., Rádis-Baptista G., Araújo T.G.d. (2023). Preparation of PLGA Nanoparticles Loaded with the Anti-Infective Ctn[15-34] Peptide for Antifungal Application. Braz. Arch. Biol. Technol..

[124] Marinelli L., Ciulla M., Ritsema J.A.S., van Nostrum C.F., Cacciatore I., Dimmito M.P., Palmerio F., Orlando G., Robuffo I., Grande R. (2022). Preparation, Characterization, and Biological Evaluation of a Hydrophilic Peptide Loaded on PEG-PLGA Nanoparticles. Pharmaceutics.

[125] Situ J.Q., Wang X.J., Zhu X.L., Xu X.L., Kang X.Q., Hu J.B., Lu C.Y., Ying X.Y., Yu R.S., You J. (2016). Multifunctional SPIO/DOX-loaded A54 Homing Peptide Functionalized Dextran-g-PLGA Micelles for Tumor Therapy and MR Imaging. Sci. Rep..

[126] Tondeur E.G.M., Voerman J.S.A., Geleijnse M.A.A., van Hofwegen L.S., van Krimpen A., Koerner J., Mishra G., Song Z., Schliehe C. (2023). Sec22b and Stx4 Depletion Has No Major Effect on Cross-Presentation of PLGA Microsphere-Encapsulated Antigen and a Synthetic Long Peptide In Vitro. J. Immunol..

[127] Ma W., Chen M., Kaushal S., McElroy M., Zhang Y., Ozkan C., Bouvet M., Kruse C., Grotjahn D., Ichim T. (2012). PLGA nanoparticle-mediated delivery of tumor antigenic peptides elicits effective immune responses. Int. J. Nanomed..

[128] Mahmoud M.Y., Steinbach-Rankins J.M., Demuth D.R. (2019). Functional assessment of peptide-modified PLGA nanoparticles against oral biofilms in a murine model of periodontitis. J. Control. Release.

[129] Jiang T., Yu X., Carbone E.J., Nelson C., Kan H.M., Lo K.W. (2014). Poly aspartic acid peptide-linked PLGA based nanoscale particles: Potential for bone-targeting drug delivery applications. Int. J. Pharm..

[130] Kostadinova A.I., Middelburg J., Ciulla M., Garssen J., Hennink W.E., Knippels L.M.J., van Nostrum C.F., Willemsen L.E.M. (2018). PLGA nanoparticles loaded with beta-lactoglobulin-derived peptides modulate mucosal immunity and may facilitate cow’s milk allergy prevention. Eur. J. Pharmacol..

[131] Pan Q., Li W., Yuan X., Rakhmanov Y., Wang P., Lu R., Mao Z., Shang X., You H. (2016). Chondrogenic effect of cell-based scaffold of self-assembling peptides/PLGA-PLL loading the hTGFbeta3 plasmid DNA. J. Mater. Sci. Mater. Med..

[132] Chen H.A., Ma Y.H., Hsu T.Y., Chen J.P. (2020). Preparation of Peptide and Recombinant Tissue Plasminogen Activator Conjugated Poly(Lactic-Co-Glycolic Acid) (PLGA) Magnetic Nanoparticles for Dual Targeted Thrombolytic Therapy. Int. J. Mol. Sci..

